# Representative Conformational Sampling for Chiroptical Analysis of the Flexible Dopamine Agonist Rotigotine

**DOI:** 10.3390/ph19091351

**Published:** 2026-08-26

**Authors:** Yi-Xuan Li, Xiao-Li Zhou, Jing-Wen Xu, Chen Zhao, You-Yang Li, Hai-You Zhao, Liang-Peng Li, Xin-Xin Tan, Yu-Xiang Zhang, Bei-Bei Yang, Li Li, Li-Hui Yin

**Affiliations:** 1 State Key Laboratory of Digestive Health, Beijing Key Laboratory of Active Substances Discovery and Druggability Evaluation, Institute of Materia Medica, Chinese Academy of Medical Sciences & Peking Union Medical College, Beijing 100050, China; liyixuan@imm.ac.cn (Y.-X.L.); zhaochen@imm.ac.cn (C.Z.); zhaohaiyou@imm.ac.cn (H.-Y.Z.); liliangpeng@imm.ac.cn (L.-P.L.); tanxinxin@imm.ac.cn (X.-X.T.); zhangyuxiang@imm.ac.cn (Y.-X.Z.); yangbetty@imm.ac.cn (B.-B.Y.); 2 State Key Laboratory of Drug Regulatory Science, National Institutes for Drug Control, Beijing 102629, China; zhouxiaoli@nifdc.org.cn (X.-L.Z.); xjwcheryl@163.com (J.-W.X.); liyouyang0313@foxmail.com (Y.-Y.L.); 3 Department of Pharmaceutical Analysis, School of Pharmacy, China Pharmaceutical University, Nanjing 210009, China

**Keywords:** rotigotine, electronic circular dichroism, vibrational circular dichroism, optical rotatory dispersion, flexible molecules, conformational analysis, time-dependent density functional theory

## Abstract

**Background/Objectives**: Rotigotine is a conformationally flexible chiral dopamine agonist, but its solution-phase chiroptical behavior and conformational heterogeneity have not been systematically assessed. This study characterized its electronic circular dichroism (ECD), optical rotatory dispersion (ORD), and vibrational circular dichroism (VCD) responses and evaluated their consistency with its absolute configuration. **Methods**: ECD and ORD data were acquired for rotigotine ((*S*)-**1**) and its simplified analog (*S*)-**2** in acetonitrile and methanol, and VCD spectra were recorded in appropriate deuterated solvents. Conformational searches, Boltzmann weighting, and density functional theory/time-dependent density functional theory calculations were performed. A principal-component-analysis/torsion (PCA-Tor) workflow selected a compact but structurally representative conformer ensemble for (*S*)-**1**. **Results**: In acetonitrile, (*S*)-**1** exhibited a positive Cotton effect (CE) at 208.5 nm and a negative CE at 194.0 nm. The Boltzmann-weighted ECD spectrum reproduced the intense negative band below 200 nm and the positive band near 210 nm. The calculated VCD spectrum agreed with the signs and positions of the main experimental bands in the 1500–1100 cm^−1^ region. Experimental ORD curves of both compounds were negative in acetonitrile and methanol. The calculations reproduced the sign and overall trend, although their magnitudes were protocol- and population-sensitive. Ring puckering strongly affected the calculated ECD and ORD data, whereas side-chain flexibility expanded the accessible conformational space. **Conclusions**: The combined results support the known *S* configuration of rotigotine and demonstrate the value of representative conformational sampling for flexible chiral pharmaceuticals. This study also defines the practical utility and limitations of simplified molecular models in chiroptical analysis.

## 1. Introduction

**Figure 1:**
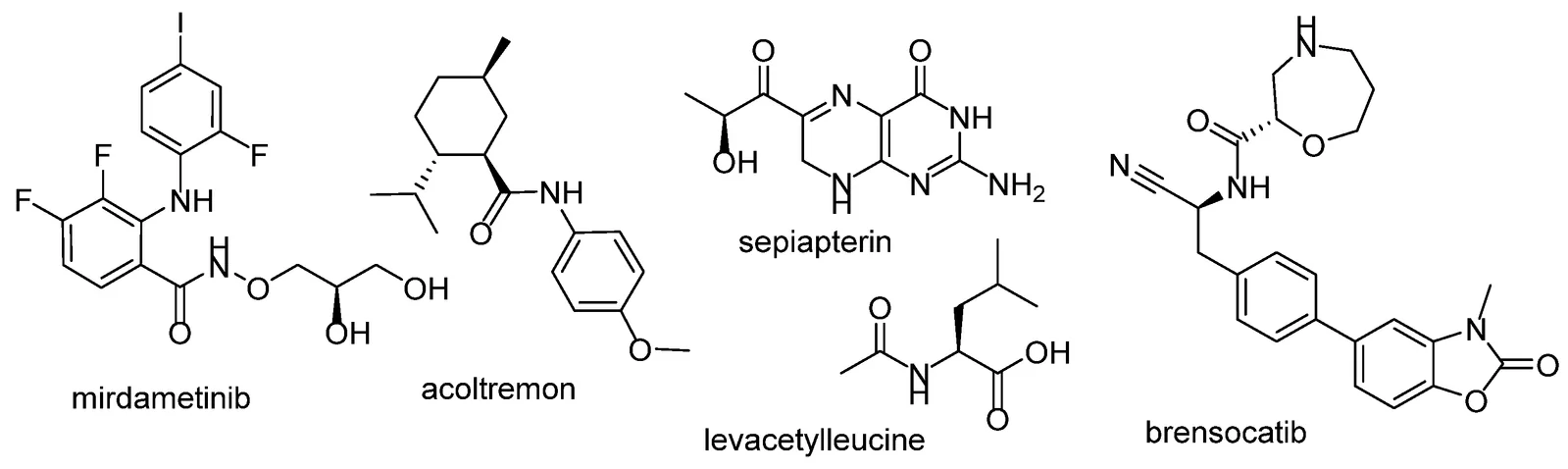
Representative conformationally flexible chiral drugs.

Stereochemistry plays a critical role in modulating the behavior of chiral drugs because distinct configurations can exhibit divergent pharmacodynamic, pharmacokinetic, and toxicological profiles [1]. Flexible molecular frameworks are common among chiral pharmaceuticals and are exemplified by recently developed conformationally mobile drugs with high bioactivity, including mirdametinib (Figure 1) [2], acoltremon [3], sepiapterin [4], brensocatib [5], and levacetylleucine [6].

Chiroptical spectroscopic methods, including electronic circular dichroism (ECD), optical rotatory dispersion (ORD), and vibrational circular dichroism (VCD), can be combined with quantum chemical calculations to determine or verify the absolute configuration of chiral drugs and thereby complement crystallographic and NMR methods in pharmaceutical characterization [7,8,9]. Nevertheless, the stereochemical analysis of flexible chiral molecules remains challenging because chiroptical responses are strongly conformer-dependent, suitable single crystals are not always available, and NMR derivatization methods often impose specific structural requirements. These factors can lead to uncertainty when the conformational ensemble is not adequately considered.

**Figure 2:**
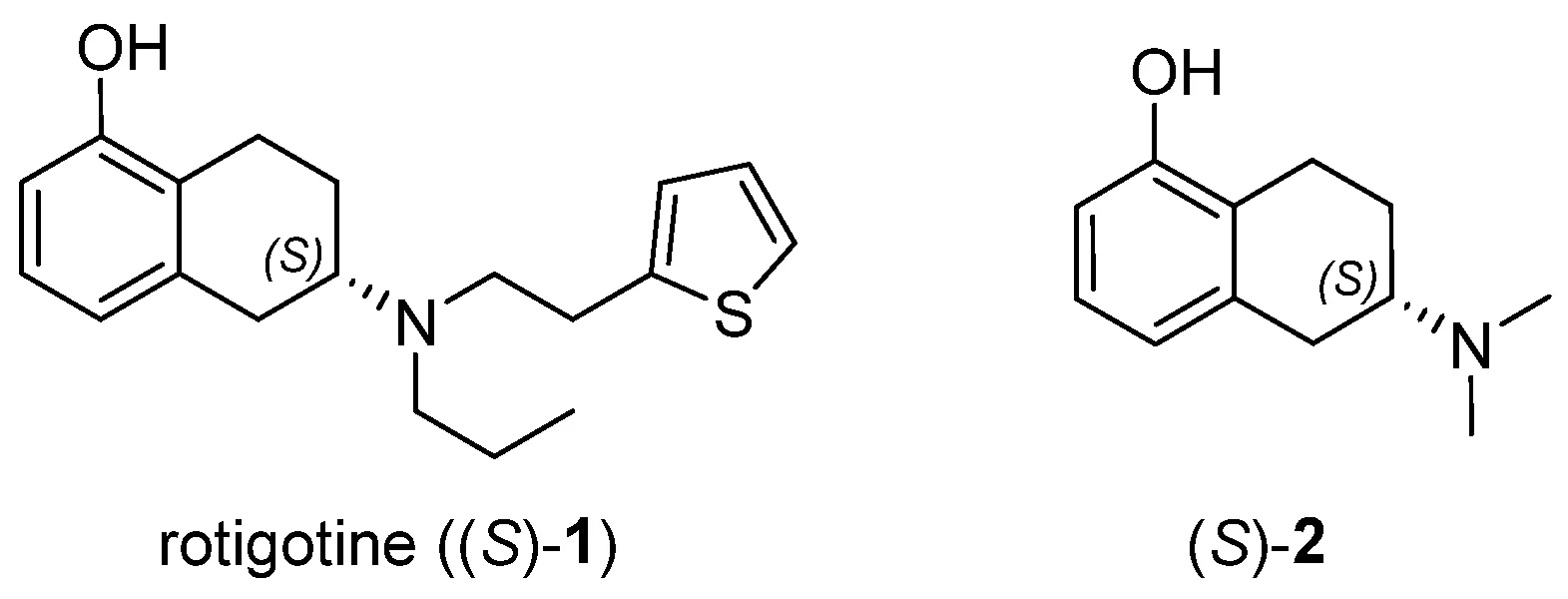
Chemical structures of rotigotine ((*S*)-**1**) and the simplified analog (*S*)-**2**.

Rotigotine ((*S*)-**1**, Figure 2) is a non-ergoline dopamine agonist with high affinity for dopamine D1–D3 receptors in the caudate putamen, which underpins its efficacy in Parkinson’s disease (PD) and restless legs syndrome [10,11]. It also shows antagonist activity at α2B-adrenergic receptors and agonist activity at 5-HT1A receptors, thereby alleviating both motor and non-motor symptoms, including depression and sleep disturbances [12,13]. Owing to its poor bioavailability, a transdermal patch formulation (Neupro^®^) was developed. The product received marketing approval from the EMA and FDA between 2006 and 2007, was temporarily withdrawn in 2008 because of crystallization problems, and was reintroduced in 2012 following formulation optimization [14]. The stereogenic center is associated with stereospecific pharmacology: the *levo*-isomer N-0437 activates postsynaptic receptors, whereas the *dextro*-isomer acts as an antagonist, necessitating the use of an enantiopure formulation [15,16,17,18].

Although the absolute configuration of rotigotine has been unambiguously confirmed by single-crystal X-ray diffraction [19], its solution-phase chiroptical behavior and the influence of conformational flexibility on its ECD, ORD, and VCD responses have not been systematically characterized. In particular, the extent to which puckering of the tetrahydronaphthalene ring and flexibility of the amino side chain affect the calculated spectra remains unclear. Our research focuses on the chiroptical behavior of chiral drugs and on the application of experimental spectroscopy and quantum-chemical calculations to the stereochemical analysis of synthetic pharmaceuticals and natural products [20,21,22]. Accordingly, we performed a combined experimental and theoretical chiroptical study of (*S*)-**1** and its simplified analog (*S*)-**2** (Figure 2).

Specifically, ECD, ORD, and VCD measurements were combined with conformational analysis and density functional theory (DFT)/time-dependent density functional theory (TDDFT) calculations to assess whether the calculated spectra reproduce the experimental signatures of the known *S* enantiomer. We further examined how puckering of the tetrahydronaphthalene core and flexibility of the amino side chain influence the chiroptical response and evaluated the extent to which (*S*)-**2** can serve as a computationally simpler model. The resulting analysis provides a practical framework for interpreting the chiroptical spectra of conformationally flexible pharmaceuticals.

## 2. Results and Discussion

### 2.1. Experimental ECD Spectra of (S)-***1*** and (S)-***2***

Cotton effects (CEs) observed in an ECD spectrum arise from the chiral perturbation of electronic transitions associated with molecular chromophores. The molecular structure of (*S*)-**1** contains an aromatic ring, a thiophene ring, a tertiary amine moiety, and a hydroxyl group. The π-systems of the benzene and thiophene rings constitute the principal chromophoric units, whereas the tertiary amino and phenolic hydroxyl groups modulate the electronic transitions and the overall chiroptical response.

**Figure 3:**
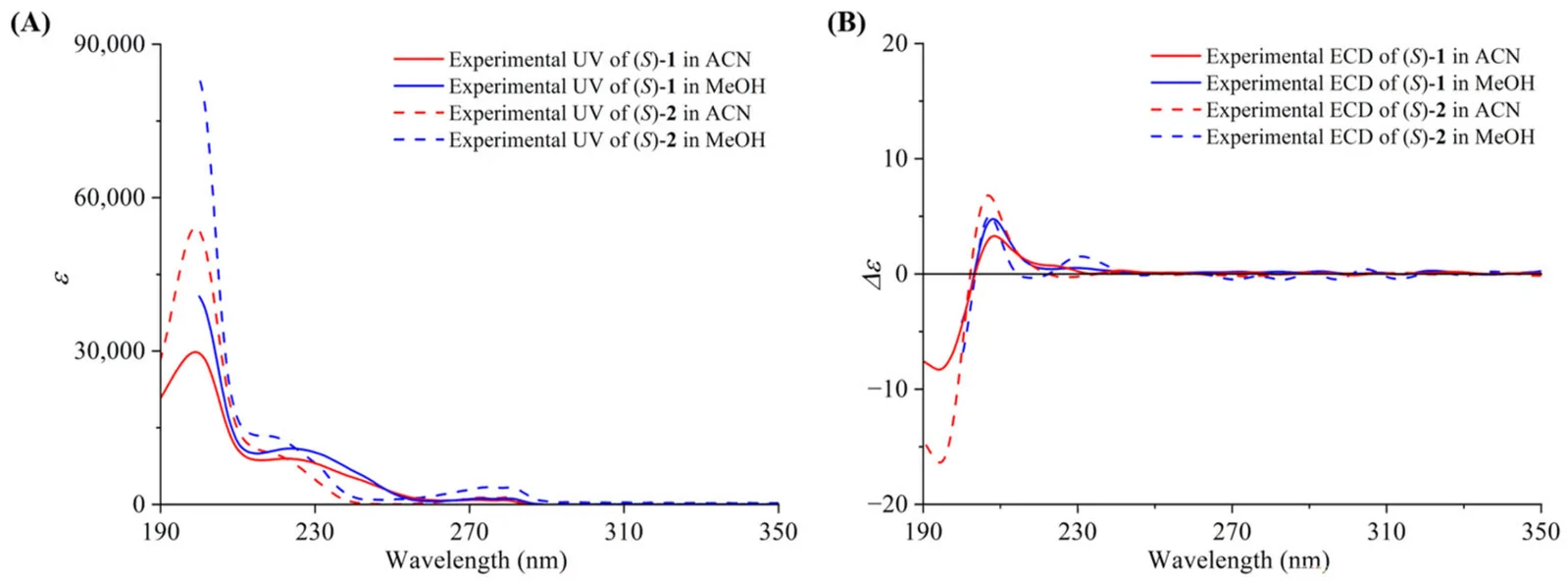
Experimental (**A**) UV and (**B**) ECD spectra of (*S*)-**1** (solid line) and (*S*)-**2** (dashed line) in acetonitrile (ACN, red) and methanol (MeOH, blue).

The UV absorption spectrum of (*S*)-**1** in acetonitrile exhibits a maximum at 199 nm, a shoulder at 222.5 nm, and a weak band at 271 nm (Figure 3A). The intense short-wavelength absorption is mainly associated with aromatic π→π* transitions, whereas the detailed electronic origins of the individual ECD bands are examined by TTDFT analysis in Section 2.5.1. The experimental ECD spectrum of (*S*)-**1** in acetonitrile displays a positive CE at 208.5 nm and a negative CE at 194 nm (Figure 3B). The UV and ECD spectra recorded in methanol show patterns closely similar to those obtained in acetonitrile.

In this study, (*S*)-**2** was used as a structurally simplified analog of (*S*)-**1** in both experimental and theoretical investigations to examine the effects of core distortion and side-chain substitution. In (*S*)-**2**, the dimethylamino group at C6 and the hydroxyl group at C1 adopt a para-like relationship across the aromatic ring, facilitating electronic communication through the conjugated π system. The experimental UV and ECD spectra of (*S*)-**2** show overall patterns similar to those of (*S*)-**1**. The similarity between the spectra of (*S*)-**1** and (*S*)-**2** indicates that the spatially remote thiophene-containing side chain has a limited effect on the principal ECD band pattern, although it affects the relative band intensities.

### 2.2. Experimental ORD Curves of (S)-***1*** and (S)-***2***

**Figure 4:**
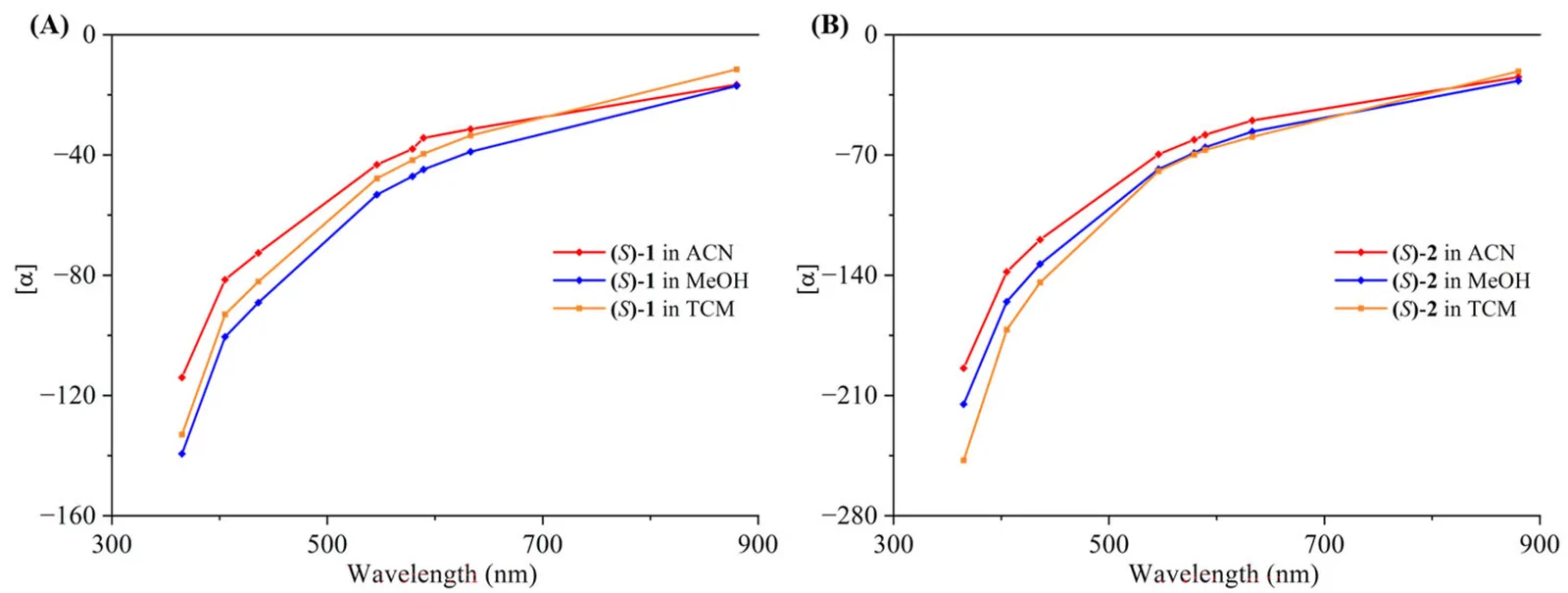
Experimental ORD of (**A**) (*S*)-**1** and (**B**) (*S*)-**2** in ACN (red), MeOH (blue), and chloroform (TCM, orange).

The experimental ORD curves of (*S*)-**1** and (*S*)-**2** were first recorded in acetonitrile and methanol. In both solvents, the two compounds were levorotatory over the measured range of 365–880 nm and exhibited similar monotonic wavelength dependence (Figure 4). Changing the solvent affected the magnitude of the specific rotation but not its sign, with larger absolute rotations observed in methanol than in acetonitrile.

The reported SOR at the sodium D line ([α]_D_) of (*S*)-**1** in methanol is −42.7 or −43.8 [23,24], while in chloroform it is +20 [25], suggesting potential mutarotation induced by the solvent. To verify this discrepancy, we measured the ORD of (*S*)-**1** in chloroform and obtained a negative [α]_D_ value (−39.6), consistent with that in methanol (Figure 4). This indicates that the literature data for chloroform may contain a typographical error.

### 2.3. Experimental VCD Spectra of (S)-***1*** and (S)-***2***

**Figure 5:**
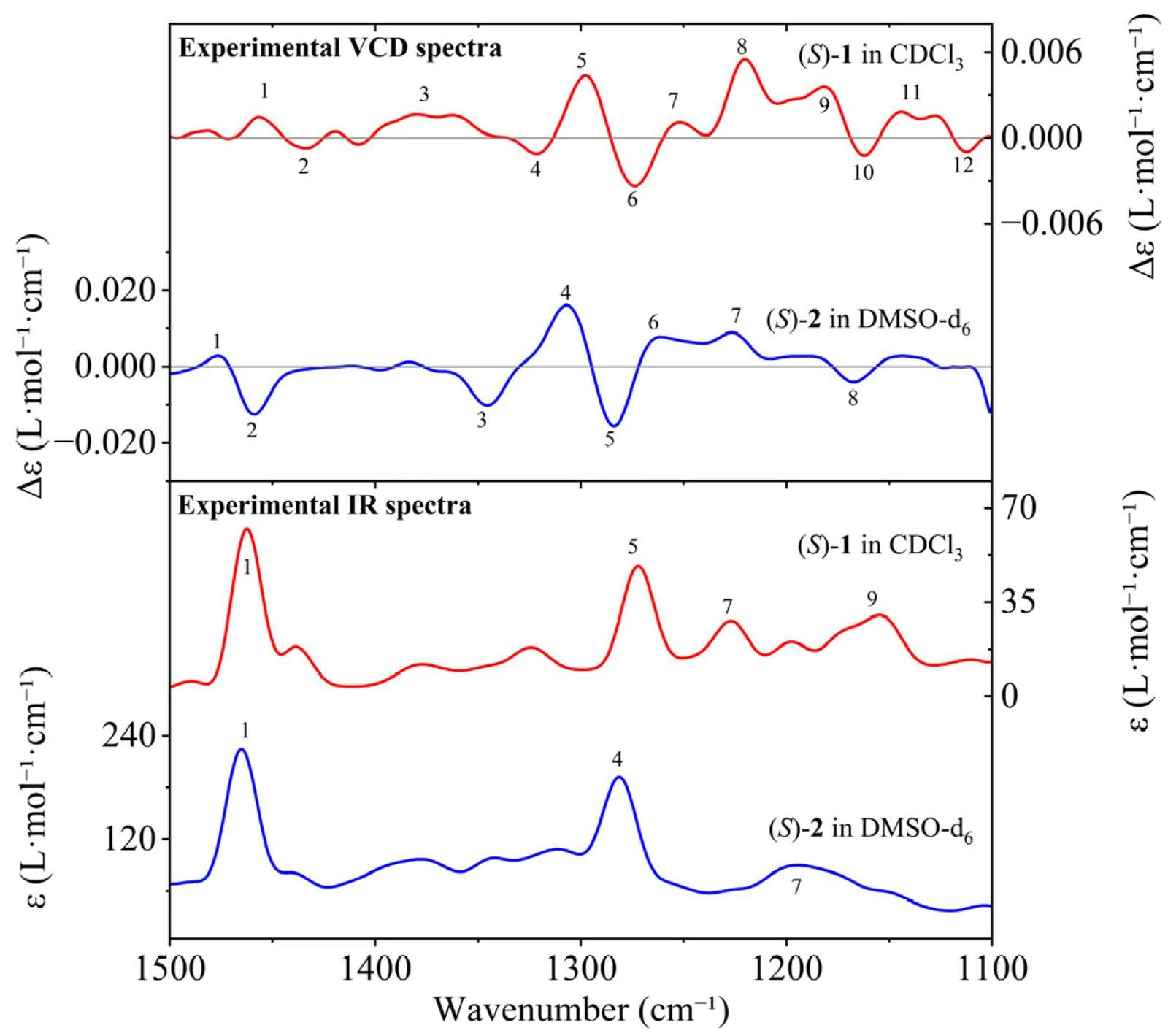
Experimental IR and VCD spectra of (*S*)-**1** (red) in CDCl_3_ and (*S*)-**2** (blue) in DMSO-d_6_.

VCD spectroscopy provides complementary vibrational information for absolute configuration analysis. Owing to their different solubility, solution-phase VCD spectra were recorded for (*S*)-**1** in deuterated chloroform (CDCl_3_) and for (*S*)-**2** in deuterated dimethyl sulfoxide (DMSO-d_6_) (Figure 5).

The experimental IR and VCD spectra of (*S*)-**1** and (*S*)-**2** displayed distinct patterns. For (*S*)-**1**, the IR band at 1463 cm^−1^ (IR-(*S*)-**1**: peak 1) was assigned mainly to aromatic and thiophene skeletal stretching coupled with aliphatic C-H deformation. The bands at 1272 and 1226 cm^−1^ (IR-(*S*)-**1**: peaks 5 and 7) were dominated by coupled phenolic C-O and tertiary-amine C-N stretching, whereas the 1155 cm^−1^ band (IR-(*S*)-**1**: peak 9) additionally involved ring C-H in-plane bending. Consistent with these assignments, the VCD spectrum displayed a negative–positive–negative CE sequence at 1321, 1287, and 1274 cm^−1^ (VCD-(*S*)-**1**, peaks 4-6), which was associated with coupled C-N/C-O stretching and C-H deformation within the chiral aminotetralin environment. The positive CEs at 1220 and 1182 cm^−1^ (VCD-(*S*)-**1**: peaks 8 and 9) were associated with related mixed stretching and in-plane bending modes.

In comparison, the IR spectrum of (*S*)-**2** showed an aromatic skeletal and CH_2_/CH_3_ deformation band at 1465 cm^−1^ (IR-(*S*)-**2**, peak 1), accompanied by a weaker aliphatic C-H bending band at 1378 cm^−1^. The absorptions at 1281 and 1195 cm^−1^ (IR-(*S*)-**2**, peaks 4 and 7), which were dominated by phenolic C-O and amine C-N stretching, also contained a contribution from aromatic C-H in-plane bending at 1195 cm^−1^. These differences were also reflected in the VCD profile, which displayed a positive–negative couplet at 1476 and 1459 cm^−1^ (VCD-(*S*)-**2**, peaks 1 and 2), followed by a negative–positive–negative sequence at 1345, 1307, and 1283 cm^−1^ (VCD-(*S*)-**2**, peaks 3–5). These features arose mainly from coupled aromatic skeletal vibrations, C-H deformation, and C-N/C-O stretching near the stereogenic center. At lower wavenumbers, the positive band at 1227 cm^−1^, negative band at 1168 cm^−1^, and positive band at 1144 cm^−1^ (VCD-(*S*)-**2**, peaks 7–9) were associated with mixed C-O/C-N stretching and ring C-H in-plane bending modes.

Solid-state IR/VCD spectra of (*S*)-**1** were also obtained using a KBr pellet (Figure S1). The solution-phase and KBr-dispersed solid-state IR/VCD spectra of (*S*)-**1** show overall consistency in the principal vibrational regions, particularly near 1463 cm^−1^ and in the 1280–1160 cm^−1^ region. The strong solution-phase IR band at 1463 cm^−1^ is retained at 1465 cm^−1^ in KBr, supporting its assignment to an intrinsic vibration of (*S)*-**1**. In contrast, the solid-state VCD spectrum displays sharper and more structured features, with changes in relative intensity and local sign distribution, especially around 1281, 1203, and 1168 cm^−1^. These spectral differences are most reasonably attributed to conformational restriction, intermolecular interactions, and modified vibrational coupling in the solid state.

### 2.4. Conformational Analysis of (S)-***1*** and (S)-***2***

#### 2.4.1. Conformational Analysis of (S)-**2**

Accurate characterization of conformational equilibrium is essential for reliable chiroptical calculations because the conformer distribution directly determines the sign, intensity, and overall profile of a Boltzmann-averaged spectrum. Individual conformers may display markedly different spectral features and, in some cases, oppositely signed dominant CEs in the same wavelength region. Consequently, even a moderately populated conformer can alter the band shape or partially cancel the contribution of the dominant conformer. Detailed conformational analysis is therefore indispensable for theoretical prediction and interpretation of chiroptical spectra.

**Figure 6:**
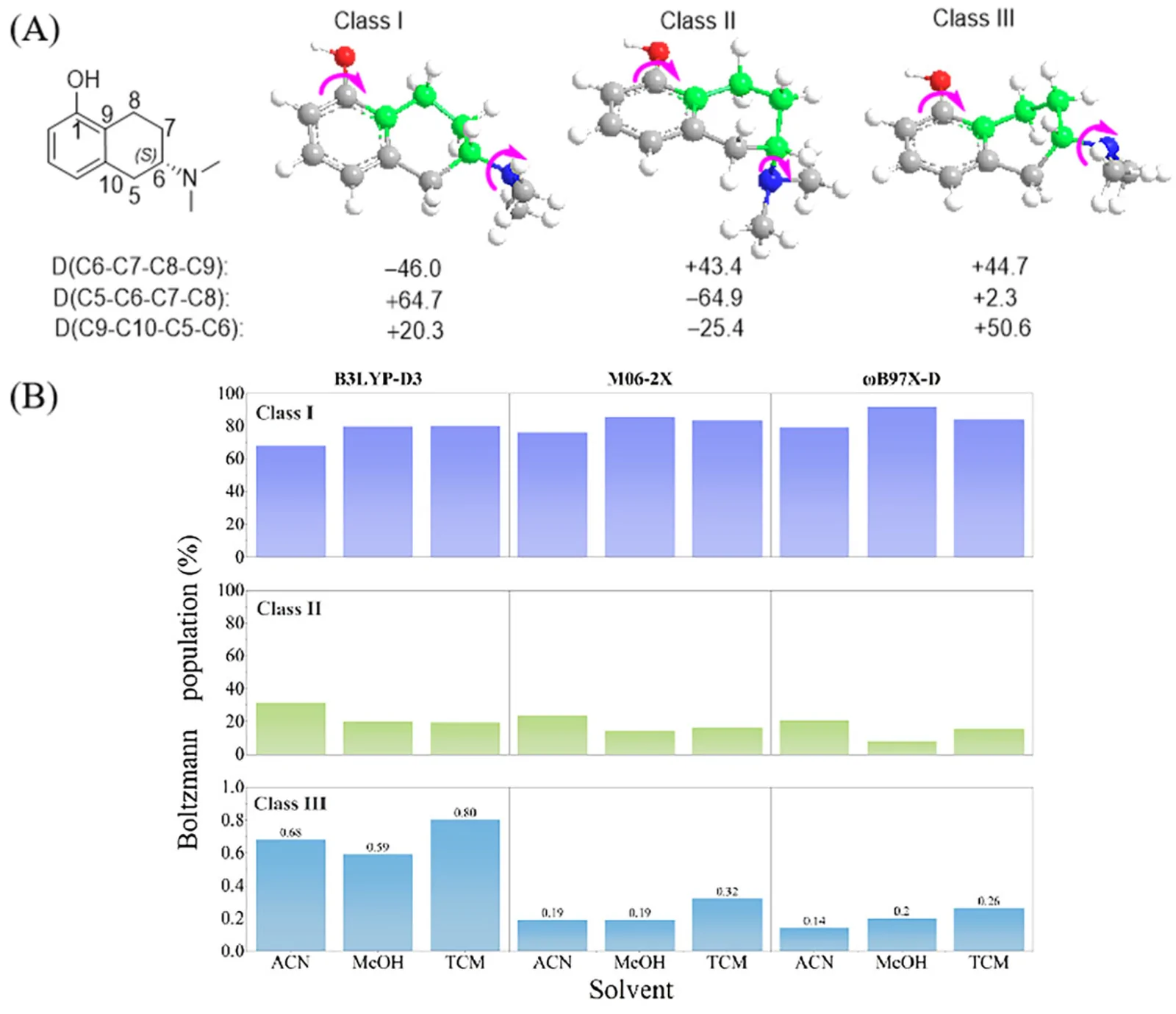
(**A**) Representative conformational variations of (*S*)-**2** arising from ring puckering and rotations of the hydroxyl group and dimethylamino moiety. Magenta arrows indicate rotation about the relevant single bonds. (**B**) Boltzmann population distributions of the three principal conformational classes of (*S*)-**2** in acetonitrile, methanol, and chloroform, calculated using the B3LYP-D3/6-311G(d,p) (B3LYP-D3), M06-2X/TZVP (M06-2X), and ωB97X-D/TZVP (ωB97X-D) methods. The Class III distributions are shown on an expanded y-axis scale of 0–1.0% for clarity.

To elucidate the conformational variation arising from ring puckering, the conformational equilibrium of (*S*)-**2** was systematically investigated. Seventeen conformers were identified through an extensive conformational search (Table S1) and classified into three categories according to the overall puckering pattern of the tetrahydronaphthalene ring (Figure 6A). In both Class I and Class II conformers, the cyclohexane moiety adopts a half-chair conformation; however, the two classes differ in the direction of ring puckering. The diagnostic dihedral angle D(C6-C7-C8-C9) is approximately −46° for Class I and +43° for Class II. The opposite signs indicate reversed puckering orientations and alter the relative spatial arrangement of the aromatic chromophore and the dimethylamino group. Consistent with this structural difference, representative Class I and Class II conformers show pronounced differences in their calculated ECD spectra and may exhibit oppositely signed CEs in the short-wavelength region rather than only changes in band intensity or position. Class III conformers adopt a higher-energy, strongly distorted envelope-like ring geometry. Although D(C6-C7-C8-C9) is approximately +45°, similar to Class II, the overall puckering pattern distinguishes Class III from the half-chair family and produces a distinct ECD response. Within each class, additional conformational variation arises mainly from the orientations of the dimethylamino group and the hydroxyl proton. These local changes do not alter the ring-puckering classification but can further modulate the intensity, position, and detailed line shape of the calculated ECD bands.

The Boltzmann populations of the 17 conformers were evaluated using three different computational protocols. Because of the molecular flexibility of (*S*)-**2** and the small Gibbs free-energy differences among many low-lying conformers, a broad conformational ensemble is thermally accessible, and the individual populations of most conformers are below 10%. Nevertheless, the predicted Boltzmann distribution is sensitive to the computational protocol, and different parameter combinations produce appreciable variations in the relative conformer populations. When the conformers are grouped according to their ring-puckering patterns, Class I remains the predominant family under all conditions examined and accounts for approximately 68–92% of the equilibrium population, whereas Class III contributes only approximately 0.2–0.7% (Figure 6B, Table S1). Inclusion of an empirical dispersion correction generally increases the relative population of Class I while decreasing that of Class II. Because Class I and Class II conformers can generate substantially different and, in some spectral regions, oppositely signed ECD responses, method-dependent changes in their relative populations directly affect the sign, intensity, and overall profile of the final Boltzmann-weighted spectrum. Theoretical chiroptical spectra should therefore be evaluated using the complete accessible conformational ensemble rather than the lowest-energy conformer alone.

#### 2.4.2. Conformational Analysis of (S)-**1**

Owing to puckering of the tetrahydronaphthalene ring and the flexibility of the *N*-propyl-2-(thiophen-2-yl)ethylamino side chain, (*S*)-**1** has a substantially larger conformational space than the truncated model compound (*S*)-**2**. The six torsional degrees of freedom used to describe the side-chain flexibility, together with their structural definitions and angular distributions, are summarized in Figures S2–S4 and Table S2. The initial MOE search generated 1758 conformers, which served as the starting pool for a study-specific hybrid coordinate-PCA/torsional-descriptor (PCA-Tor) workflow. Unlike xTB [26], which primarily provides rapid approximate geometry refinement and energy ranking, the PCA-Tor workflow selects representatives according to geometric and torsional diversity, thereby improving conformational-space coverage beyond that ensured by energy ranking alone. This consideration is particularly important in chiroptical analysis because conformers with the same absolute configuration may exhibit markedly different spectra and contribute unequally to Boltzmann-averaged responses [27]. Clustering-based conformer prioritization has also been shown to reduce redundant DFT re-optimization [28]. PCA-Tor-based selection was therefore used to reduce the conformer pool while retaining its principal structural diversity.

**Figure 7:**
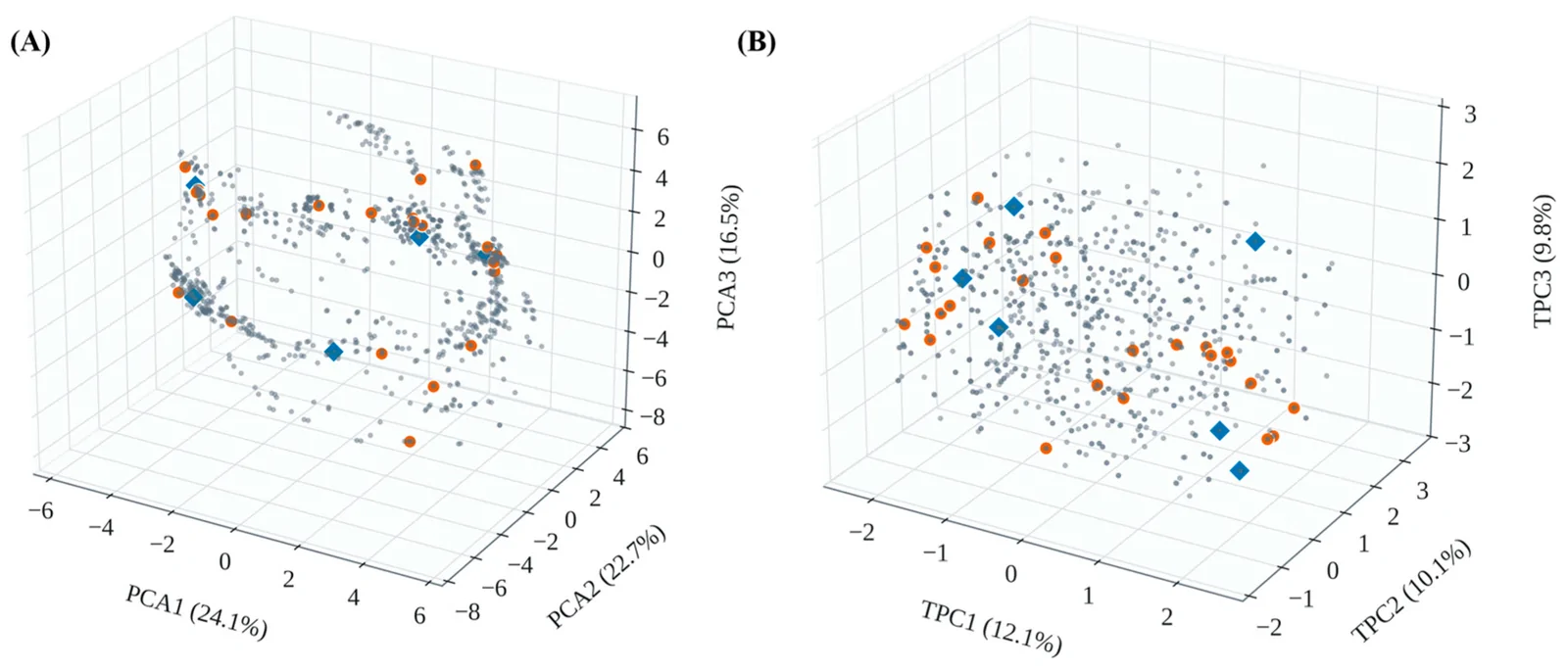
Low-dimensional visualization of the PCA-Tor-selected conformers of (*S*)-**1**. (**A**) Coordinate-PCA projection of the 947 prefiltered conformers, in which PCA1-PCA3 together account for 63.3% of the coordinate variance. (**B**) Torsion-PCA projection derived from the sine/cosine encodings of T1-T6, in which TPC1-TPC3 together account for 32.0% of the torsional variance. In both panels, the gray points represent the prefiltered conformer pool (*n* = 947), the blue diamonds denote the six low-energy anchor conformers (*n* = 6), and the amber circles indicate the 24 additional representative conformers (*n* = 24). These low-dimensional plots are shown for visualization purposes only; the actual conformer selection was performed in the full standardized 21-dimensional PCA-Tor descriptor space.

After filtering by initial relative energy and steric quality, 947 conformers were retained (Figure 7). PCA of the aligned Cartesian coordinates was used to describe the principal variations in overall molecular geometry. Six molecule-specific dihedral angles (T1–T6) were introduced to capture the principal local torsional variations in the flexible molecular framework. Each torsion was represented by its sine and cosine components to preserve angular periodicity and avoid boundary discontinuities [29,30,31] (Figure S4). Because PCA1–PCA9 accounted for 90.76% of the coordinate variance, their scores were retained as global geometric descriptors (Figure S5 and Table S3). The complete pairwise projections and one-dimensional distributions of PCA1–PCA9 further illustrate the distributions of the prefiltered and selected conformers along the retained coordinate components (Figure S6). The resulting nine coordinate-PCA scores and twelve torsional variables were standardized to define a 21-dimensional PCA-Tor descriptor space.

A 30-conformer working set was predefined to maintain a tractable subsequent DFT workload. On the basis of conformer-pair considerations in chiroptical analysis [32,33], six low-initial-energy conformers were retained as three structurally complementary anchor pairs; the members of each pair differed by less than 0.06 kcal/mol at the initial MOE level but exhibited distinct geometric or torsional characteristics. The remaining 24 representatives were selected by K-means partitioning of the standardized descriptor space [34,35], with regional allocation reflecting conformer occupancy while preserving representation of sparsely populated regions. The procedure was repeated three times to reduce sensitivity to cluster initialization. The reproducibility of the pair-aware selection workflow was additionally evaluated using the consensus sensitivity analysis shown in Figure S7. Coordinate- and torsion-PCA projections confirmed that the selected conformers were distributed across the principal regions of the prefiltered conformer pool (Figure S8); these projections were used only for visualization, whereas selection was performed in the full 21-dimensional space.

Compared to direct DFT treatment of all 947 prefiltered conformers, the PCA-Tor workflow reduced the number of structures submitted to quantum-chemical calculations to 30 while retaining broad conformational coverage. All 30 selected conformers were subjected to geometry optimization, frequency analysis, and TDDFT-ECD calculations. Gaussian input validation and Boltzmann populations are summarized in Tables S4–S6, and Cartesian coordinates of the main conformers are listed in Table S7.

### 2.5. Theoretical Prediction of the Chiroptical Spectra of (S)-***1*** and (S)-***2***

#### 2.5.1. ECD Simulation

Accurate theoretical ECD spectra require both representative conformer populations and an appropriate electronic-structure method. In the present study, the PCA-Tor workflow was employed to construct a compact yet conformationally representative ensemble of (*S*)-**1** for Boltzmann-weighted ECD calculations. B3LYP, Cam-B3LYP and several frequently used functionals were evaluated to assess the sensitivity of the calculated spectra and to improve consistency with the experimental spectra.

**Figure 8:**
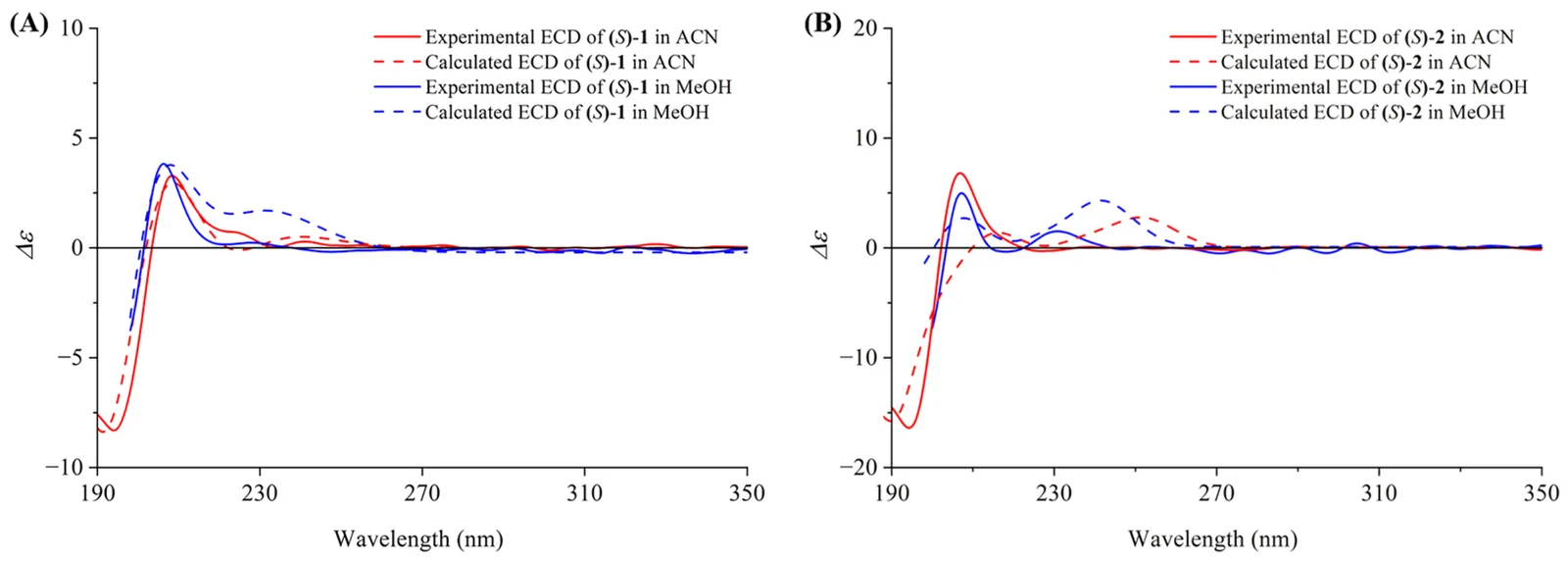
Comparison of the Boltzmann-weighted calculated (dashed line) and experimental (solid line) ECD spectra of (*S*)-**1** and (*S*)-**2** in acetonitrile and methanol. (**A**) (*S*)-**1**, calculated at SMD/solvent/Cam-B3LYP/Aug-cc-pVDZ//B3LYP-D3/6-31G(d,p), σ = 0.38 eV; (**B**) (*S*)-**2**, calculated at SMD/solvent/B3LYP/6-311+G(d,p)//B3LYP-D3/6-311G(d,p), σ = 0.25 eV. Red and blue curves denote the spectra in acetonitrile and methanol, respectively.

The Boltzmann-weighted TDDFT-ECD spectrum of (*S*)-**1** derived from the PCA-Tor-selected conformers reproduced the principal experimental features, including the intense negative band below 200 nm, the positive CE near 210 nm, and weaker features in the 230–250 nm region (Figure 8A).

The results showed that a reduced but representative ensemble could reproduce the experimental spectra. Meanwhile, the predicted ECD curves of (*S*)-**2** matched the experimental data well (Figure 8B).

To clarify the electronic origins of the calculated ECD bands, the excited states and associated hole–electron distributions [36] of (*S*)-**2** were analyzed. The results indicate that different spectral regions contain distinct types of electronic excitation. Because the largest conformer-dependent differences were associated with the fifth singlet excited state (S5), the following discussion focuses on this state.

**Figure 9:**
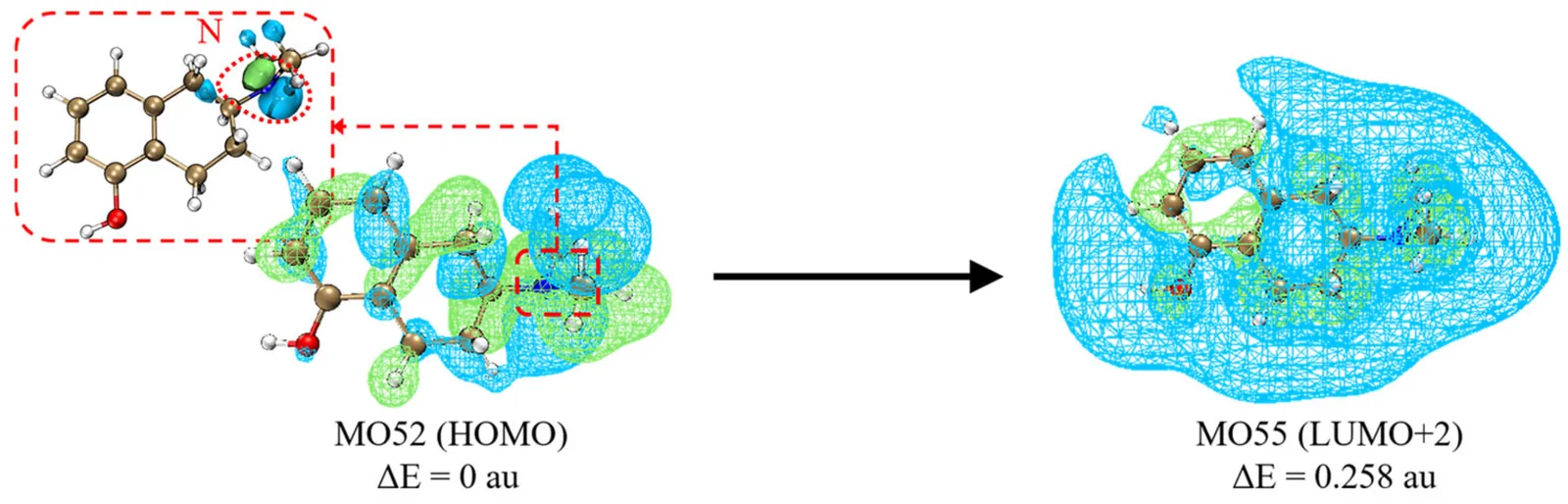
Molecular orbitals involved in the dominant S5 excitation of (*S*)-**2**. The HOMO and LUMO+2 isosurfaces were plotted at an isovalue of 0.01. The enlarged N-centered region and its correspondence with the main orbital representation (red dashed outlines and arrows). The green and blue isosurfaces represent the positive and negative regions (phases) of the molecular orbital wavefunction, respectively, and the electronic excitation from MO52 (HOMO) to MO55 (LUMO+2) (black arrow) are shown for clarity.

S5 is dominated by the MO52→MO55 excitation (Figure 9), with a configuration contribution of 79.54% (Table S8). The donor orbital MO52 (HOMO) is primarily localized on the nonbonding lone pair of the tertiary amine nitrogen, and N contributes 53.40% (Table S9) to the hole distribution. Because the orbital extends only slightly into the adjacent bonding region, MO52 is assigned as a nitrogen-centered lone-pair orbital. In contrast, the acceptor MO55 (LUMO+2) has an approximately spherical and highly diffuse outer isosurface surrounding the molecular framework, with little directionality or angular nodal character, consistent with a molecular 3s-like Rydberg orbital. Its slight ellipsoidal distortion relative to an ideal atomic 3s orbital probably arises from the non-spherical molecular potential. Moreover, the contribution of each non-hydrogen atom to the excited-electron distribution is below 10%, indicating that the excited electron is broadly distributed outside the molecular framework rather than localized on a specific atom or bond.

The hole–electron descriptors further support this assignment. The small centroid separation (Table S10, *D* = 0.387 Å) and negative *t* index (*t* = −0.997 Å) exclude pronounced long-range charge transfer. The relatively low overlap index (*S*r = 0.387) reflects reduced overlap between the localized hole and the diffuse electron distribution. In addition, the positive Δ*σ* value of 1.443 Å indicates that the electron spatial extent (*σ*_e_ = 3.193 Å) is substantially larger than the hole spatial extent (*σ*_h_ = 1.750 Å), whereas the markedly lower electron-delocalization index (EDI = 3.32) than hole-delocalization index (HDI = 17.74) confirms greater delocalization of the electron distribution. Collectively, these results support assignment of MO52→MO55 as a Rydberg excitation from the nitrogen lone-pair orbital to a 3s-like Rydberg orbital rather than a localized valence excitation or a long-range charge-transfer excitation.

#### 2.5.2. SOR and ORD Simulation

[α]_D_ is one of the most widely reported chiroptical parameters for characterizing optically active compounds and can be predicted with reasonable accuracy when an appropriate quantum-chemical protocol is available [37]. However, accurate SOR and ORD predictions for flexible molecules remain difficult because the calculated values are highly sensitive to molecular geometries and conformations [38].

**Figure 10:**
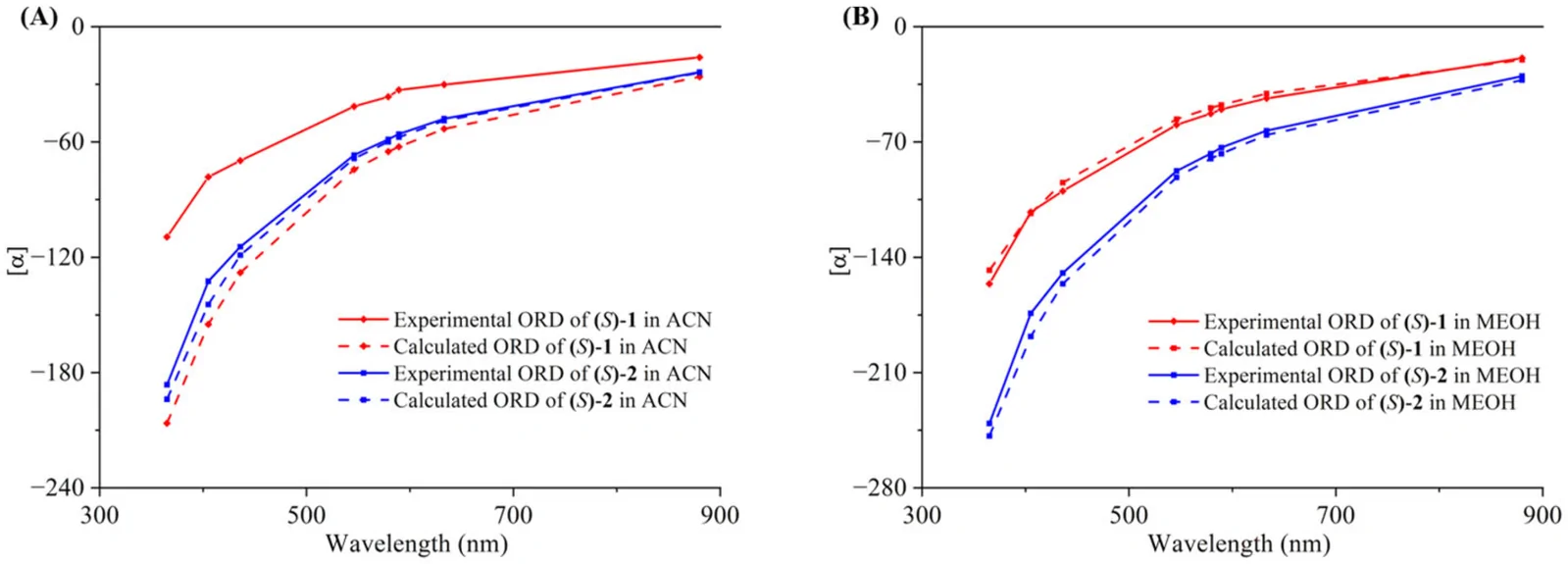
Comparison of the calculated (dashed line) and experimental (solid line) ORD curves of (*S*)-**1** (red) and (*S*)-**2** (blue) in (**A**) acetonitrile and (**B**) methanol. (*S*)-**1** calculated at the SMD/solvent/Cam-B3LYP/Aug-cc-pVDZ//ωB97X-D/TZVP level; (*S*)-**2** calculated at the SMD/solvent/ωB97X-D/Aug-cc-pVDZ//ωB97X-D/TZVP level.

For (*S*)-**1** and (*S*)-**2**, SOR and ORD calculations exhibited excellent agreement with the experimental data (Figure 10). SOR values at the monitored wavelength were negative in acetonitrile and methanol, with significantly larger absolute magnitudes in methanol compared to acetonitrile. As to (*S*)-**1**, the accuracy of the theoretical results is mainly attributed to the correct analysis of PCA-Tor-selected conformers. Comparable agreement was also observed in chloroform, where the calculated ORD curves of (*S*)-**1** and (*S*)-**2** closely reproduced the corresponding experimental profiles (Figure S9).

#### 2.5.3. VCD Simulation

**Figure 11:**
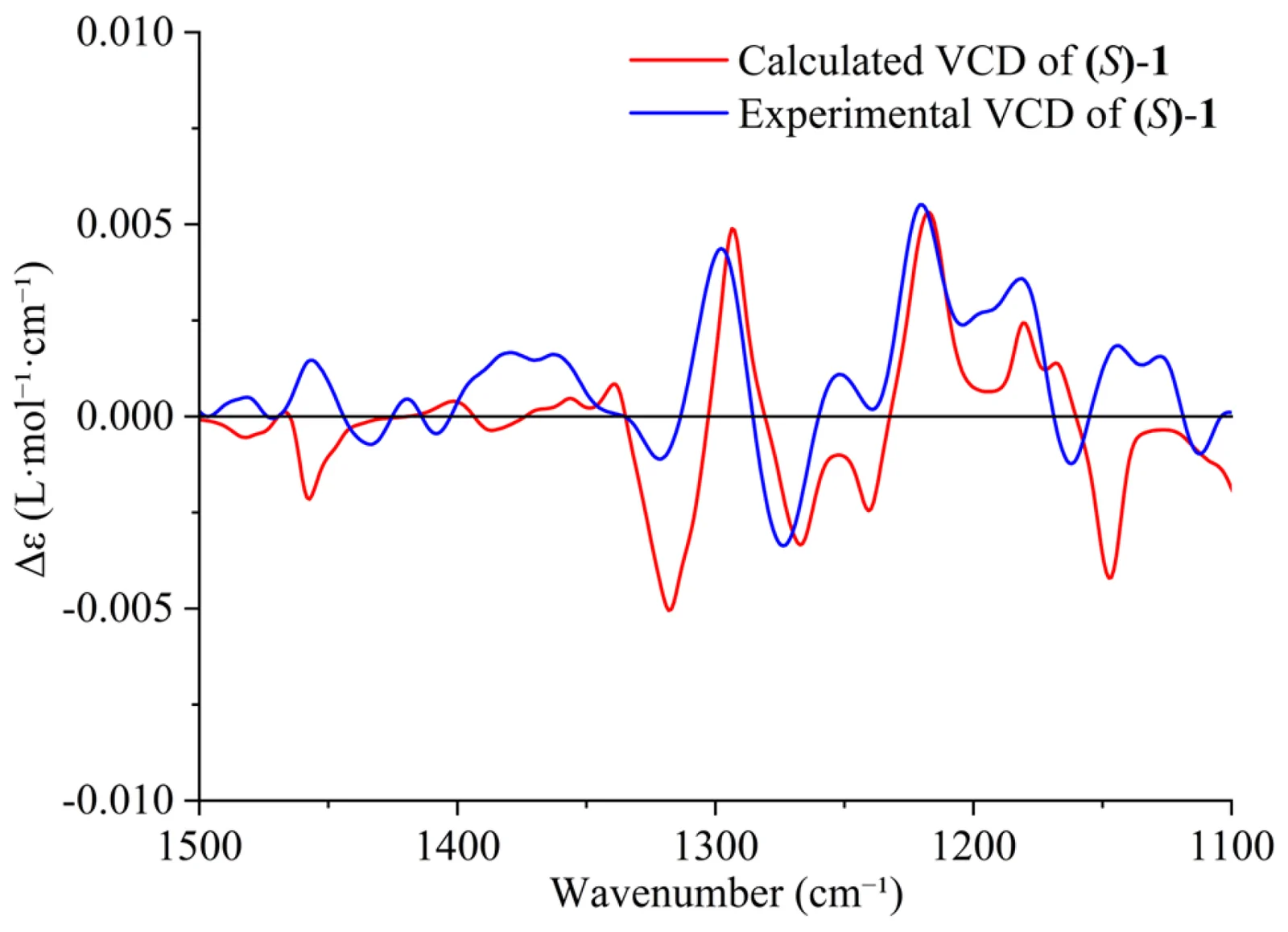
Comparison of the calculated (red) and experimental (blue) VCD spectra of (*S*)-**1** in CDCl_3_. Calculations were performed at the SMD/CDCl_3_/B3LYP-D3/6-311++G(2d,p) level.

To provide complementary support for the stereochemical analysis, the calculated VCD spectrum of (*S*)-**1** was compared with the experimental solution-phase spectrum recorded in CDCl_3_. In the 1500–1100 cm^−1^ region, the calculated spectrum reproduced the positions and signs of the principal experimental bands (Figure 11). This agreement supports the known S configuration of (*S*)-**1** and indicates that the selected full-molecule VCD protocol captures the major experimental features.

## 3. Materials and Methods

### 3.1. General Information

(*S*)-**1** (purity: 99.9%) and (*S*)-**2** (purity: 99.5%) were obtained from Tianjin Alta Scientific Co., Ltd. and China National Standard Pharmaceutical Co. Ltd., respectively. ORD measurements were performed using an Anton Paar MCP5500 polarimeter with a 0.5 dm cell and acetonitrile, methanol, or chloroform as the solvent. ECD spectra were recorded at room temperature using a J-1500 spectrometer and spectroscopic-grade solvents. The ECD measurements were performed with a path length of 0.1 cm, a scanning speed of 100 nm/min, a bandwidth of 1 nm, and an accumulation of two scans. VCD spectra were obtained using an FVS-6000 spectrometer at a resolution of 4 cm^−1^ over the range of 2000–850 cm^−1^. Solution-phase spectra were measured in spectroscopic-grade deuterated solvents, and the solid-phase spectrum was recorded using a KBr pellet.

### 3.2. Computational Section

Conformational searches were performed with MOE software (2015.9, Chemical Computing Group Inc., Montreal, QC, Canada) using the MMFF94 force field, and conformers within an energy window of 7 kcal/mol were regarded as the initial ensemble. DFT calculations were carried out using Gaussian 16 Rev B.01 [39]. Boltzmann populations at 298.15 K and 1 atm were calculated from the corresponding Gibbs free energies. The SMD model was applied for solvation, and PCM was also examined where indicated. ECD and ORD calculations were performed at the B3LYP-D3/6-311+G(d,p), ωB97X-D/6-311+G(d,p), ωB97X-D/TZVP, or Cam-B3LYP/Aug-cc-pVDZ levels. For the VCD simulation, calculations were performed at the SMD/CDCl_3_/B3LYP-D3/6-311++G(2d,p) level. SpecDis 1.71 software [40,41] was used to generate Boltzmann-averaged ECD and VCD spectra and averaged optical-rotation data. The hole–electron analyses were carried out using Multiwfn [42].

PCA-Tor-based conformer selection of (*S*)-**1** was performed as follows: of the 1758 conformers generated by MOE, 947 conformers with initial relative energies of ΔE ≤ 5.0 kcal/mol and no detected steric clashes were retained. The first nine coordinate-PCA components were combined with sine/cosine encodings of six key torsion angles, which avoid discontinuities arising from angular periodicity [43], to construct a standardized 21-dimensional PCA-Tor descriptor. Six low-initial-energy, structurally complementary conformers were retained as three anchor pairs, and the remaining candidates were partitioned into operational regions of conformational space by K-means clustering [33]. Twenty-four additional conformers were selected using a density-stratified procedure that considered regional occupancy, initial energy, and structural redundancy. The final representatives were identified by consensus across repeated K-means runs using predefined random seeds, yielding the 30-conformer set. PCA1-PCA3 and TPC1-TPC3 were used solely for visualization and did not define the selection space. The selected conformers were subsequently optimized and subjected to DFT calculations.

### 3.3. Experimental Section

#### 3.3.1. ECD Spectra

The ECD spectra of (*S*)-**1** and (*S*)-**2** were measured in acetonitrile and methanol. For both compounds, the sample concentration was 0.10 mg/mL in acetonitrile and 0.05 mg/mL in methanol. Baseline correction was performed by subtracting the spectrum of the corresponding solvent recorded under identical conditions. The final spectra were processed using the instrument software and converted to molar circular dichroism (Δε) when required.

#### 3.3.2. ORD Data

ORD data of (*S*)-**1** and (*S*)-**2** were recorded at room temperature in spectroscopic-grade methanol, acetonitrile and chloroform. Each compound was dissolved in the corresponding solvent to give a final concentration of 0.1 g/100 mL. The reported values at 365, 405, 436, 546, 579, 589, 633, and 880 nm are averages of three measurements.

#### 3.3.3. VCD Spectra

For (*S*)-**1**, solution-state VCD was measured in CDCl_3_ at 10 mg/mL using a BaF_2_ cell with a path length of 150 μm, and solid-state VCD was measured using a KBr pellet containing 0.58 mg of sample. For (*S*)-**2**, solution-state VCD was measured in DMSO-d_6_ at 35 mg/mL using a BaF_2_ cell with a path length of 150 μm. Absorbance and VCD signals were collected simultaneously, with 5000 scans for (*S*)**-1** and 2000 scans for (*S*)-**2**. Background spectra of the corresponding solvent or KBr matrix were recorded under identical conditions and subtracted from the sample spectra.

## 4. Conclusions

Experimental ECD, VCD, and ORD measurements were combined with conformational analysis and quantum-chemical calculations to characterize the chiroptical behavior of (*S*)-**1** and (*S*)-**2**. The ECD spectra of the two compounds showed similar overall band patterns, indicating that the simplified analog retains the principal electronic-chiroptical features of the tetrahydronaphthalene core, although the thiophene-containing side chain influences band intensities and substantially enlarges the accessible conformational space of (*S*)-**1**. Analysis of (*S*)-**2** demonstrated that ring puckering strongly affects the sign and profile of the calculated ECD response. For (*S*)-**1**, the PCA-Tor workflow reduced 947 prefiltered conformers to 30 representative structures, and the Boltzmann-weighted spectrum reproduced the main experimental ECD features. The calculated full-molecule VCD spectrum also reproduced the signs and positions of the principal experimental bands and provided complementary support for the S configuration. Overall, this study provides a practical conformational-sampling and chiroptical-analysis framework for flexible pharmaceuticals and clarifies both the utility and the limitations of structural simplification.

## Data Availability

The original contributions presented in the study are included in the article and Supplementary Materials; further inquiries can be directed to the corresponding author.

## Supplementary Materials

The following supporting information can be downloaded at https://www.mdpi.com/article/10.3390/ph19091351/s1: Figure S1: Experimental IR and VCD spectra of (*S*)-**1** in KBr; Figure S2: Definition of T1–T6 torsion angles used in the PCA-Tor workflow; Figure S3: Individual structural interpretation of the six key torsion angles T1–T6; Figure S4: Torsional descriptor diagnostics; Figure S5: Coordinate-PCA descriptor diagnostics; Figure S6: Pairwise coordinate-PCA projections of PCA1–PCA9; Figure S7: Reproducibility logic of the 6+24 pair-aware PCA-Tor runner; Figure S8: Two-dimensional PCA-Tor projections of the selected rotigotine conformers; Figure S9: Comparison of the calculated and experimental ORD curves of (*S*)-**1** and (*S*)-**2** in chloroform; Table S1: Conformational classification of (*S*)-**2** and corresponding theoretical ECD spectra; Table S2: Definitions and relative importance of the six key torsions used for torsion-based descriptor construction; Table S3: Explained variance ratios of the coordinate-PCA components retained in the PCA-Tor descriptor space; Table S4: Gaussian input validation summary for the selected conformers of (*S*)-**1**; Table S5: Final ACN 30-conformer Boltzmann populations at 298.15 K; Table S6: Final MeOH 30-conformer Boltzmann populations at 298.15 K; Table S7: Cartesian coordinates of main conformers (Boltzmann population > 0.5%) of (*S*)-**1** calculated at the B3LYP/6-31G(d,p) level in acetonitrile; Table S8: Major orbital contributions, excitation energies, wavelengths, and rotatory strengths of the first seven excited states of (*S*)-**2**; Table S9: Atomic contributions of non-hydrogen atoms to the first seven excited states of (*S*)-**2**; Table S10: Hole–electron analysis parameters (D, Sr, H, Δσ, t, HDI, and EDI) for the first seven excited states of (*S*)-**2**.
