# Supplementary material for: Representative Conformational Sampling for Chiroptical Analysis of the Flexible Dopamine Agonist Rotigotine
Source: Pharmaceuticals (Basel). 2026 Aug 26;19(9):1351. doi: 10.3390/ph19091351 (PMC13610727; doi:10.3390/ph19091351)
Supplement: Supplementary file 1 [file pharmaceuticals-19-01351-s001.zip › pharmaceuticals-4517396-supplementary.pdf]

Supplementary Materials

Representative Conformational Sampling for Chiroptical Analysis of the Flexible Dopamine Agonist Rotigotine

Yi-Xuan Li [1,†], Xiao-Li Zhou [2,†] ,Jing-Wen Xu [2,,3], Chen Zhao [1], You-Yang Li [2,3], Hai-You Zhao [1], Liang-Peng Li [1], Xin-Xin Tan [1], Yu-Xiang Zhang [1], Bei-Bei Yang [1], Li Li [1,*], and Li-Hui Yin [2,*]

[1.] State Key Laboratory of Digestive Health, Beijing Key Laboratory of Active Substances Discovery and Druggability Evaluation, Institute of Materia Medica, Chinese Academy of Medical Sciences & Peking Union Medical College, Beijing 100050, China
[2.] State Key Laboratory of Drug Regulatory Science, National Institutes for Drug Control, Beijing 102629, China
[3.] Department of Pharmaceutical Analysis, School of Pharmacy, China Pharmaceutical University, Nanjing 210009, China
[*] Correspondences: annaleelin@imm.ac.cn (L. Li) ; yinlihui@nifdc.org.cn (L.-H. Yin)
[†] These authors contributed equally to this work.

Contents

Experimental IR and VCD spectra of (*S*)-**1.**	S1
Conformational classification, conformer-specific ECD spectra of (*S*)-**2**.	S2
Structural definitions and torsional descriptor diagnostics of T1–T6.	S3
Gaussian input validation, Coordinate-PCA descriptors, reproducibility analysis, and PCA-Tor conformer-space visualization.	S6
Solvent-specific Gibbs free energies and Boltzmann populations in acetonitrile and methanol.	S12
Comparison of the calculated and experimental ORD curves of (*S*)-**1** and (*S*)-**2** in chloroform.	S14
Cartesian coordinates of main conformers (Boltzmann population > 0.5%) of (*S*)-**1** in acetonitrile.	S15
Excited-state and hole–electron analyses of (*S*)-**2**.	S23

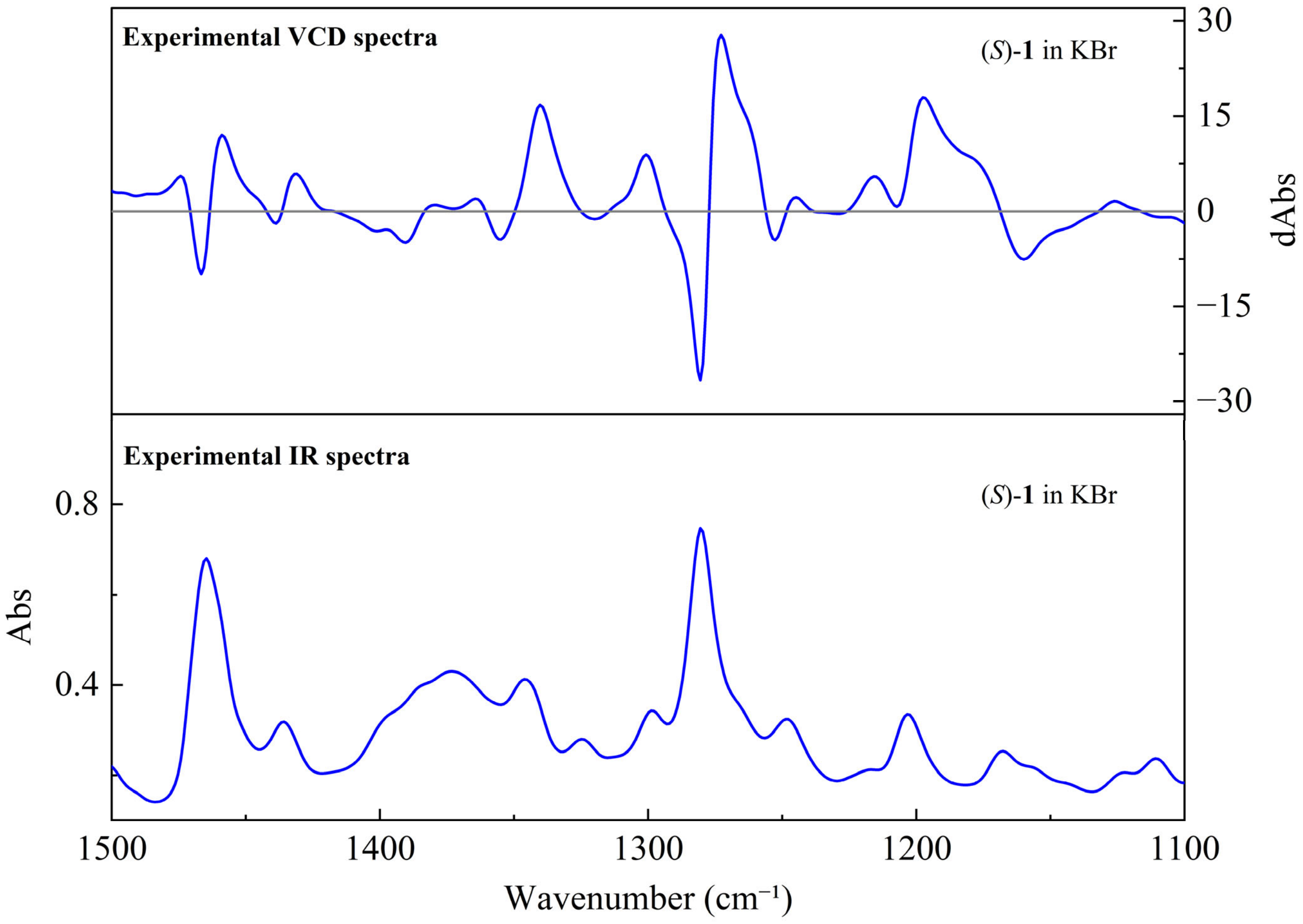

Figure S1. Experimental IR and VCD spectra of (*S*)-**1** in KBr.

Table S1. Conformational classification of (S)-**2** and corresponding theoretical ECD spectra.

Group	Conformers	Theoretical ECD spectra
Class I<br>(79.44%)	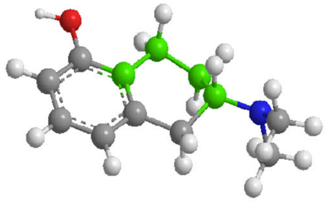 (S)-**2C1** 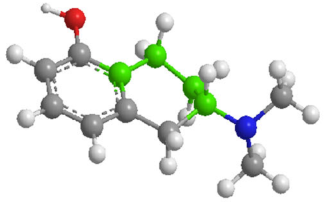 (S)-**2C2** 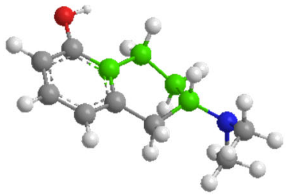 (S)-**2C3** 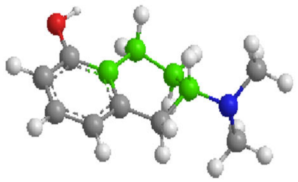 (S)-**2C4** 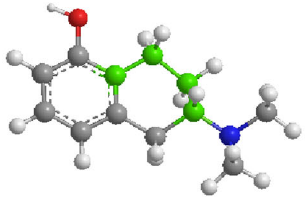 (S)-**2C8** 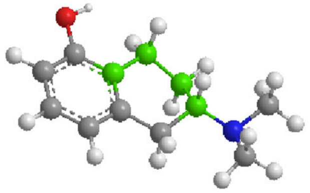 (S)-**2C13**	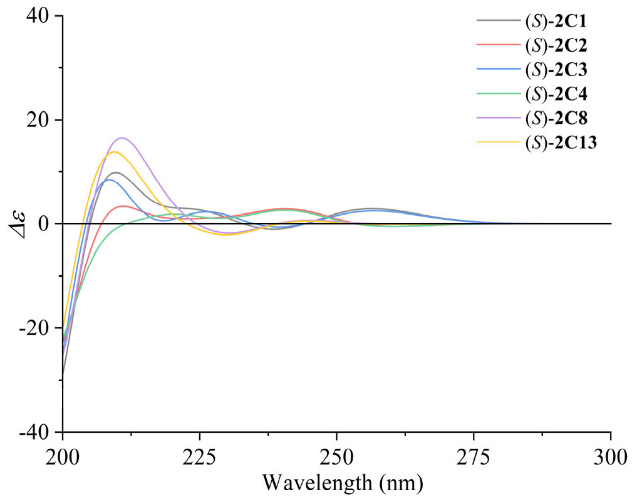
Class II<br>(19.94%)	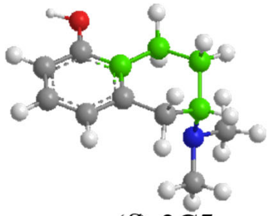 (S)-**2C5** 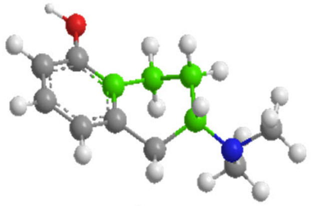 (S)-**2C6** 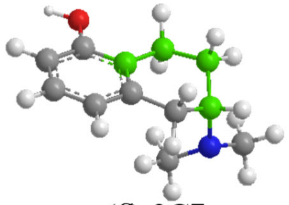 (S)-**2C7** 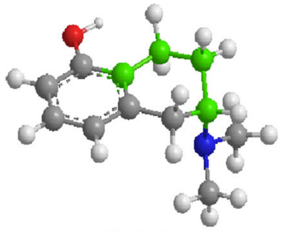 (S)-**2C10** 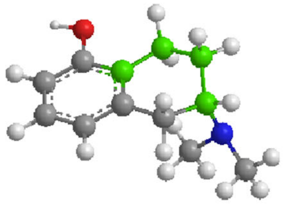 (S)-**2C11** 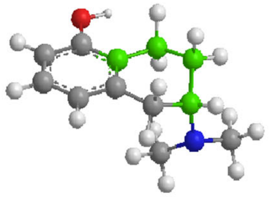 (S)-**2C14** 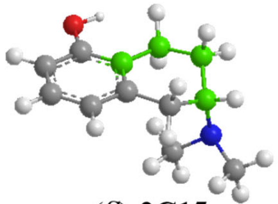 (S)-**2C15**	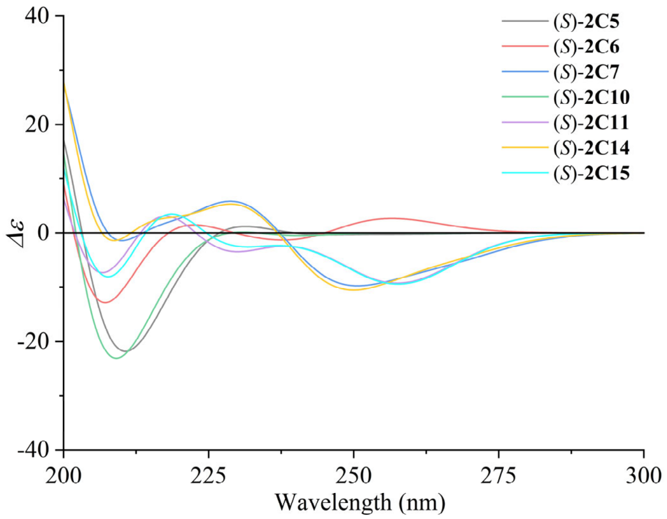

Class III<br>(0.59%)	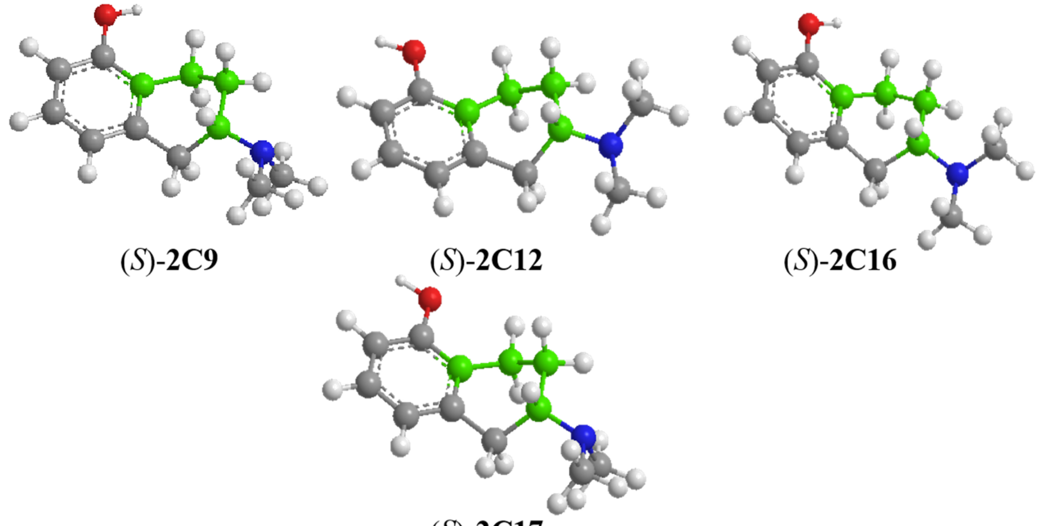<br>(*S*)-**2C9** (*S*)-**2C12** (*S*)-**2C16** (*S*)-**2C17**	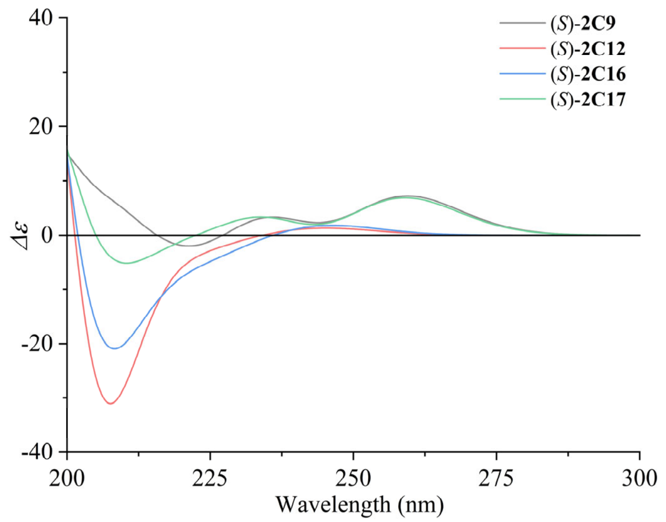

Structural definition of the six key torsions

The six torsions T1-T6 were selected to describe the major rotatable bonds connecting the aminotetralin core, tertiary amine, N-propyl substituent, and 2-thienylethyl substituent.

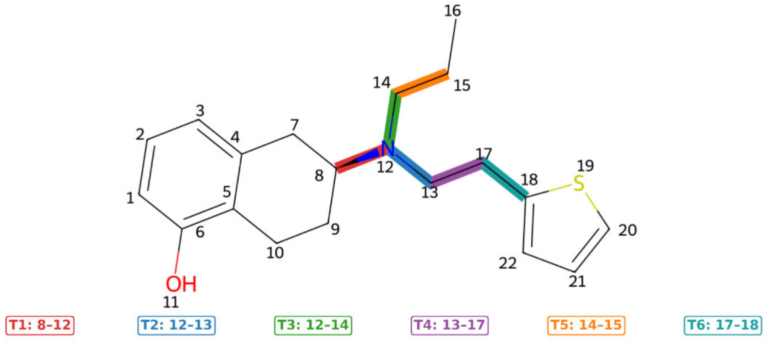

Figure S2. Definition of T1-T6 torsion angles used in the PCA-Tor workflow. (A) Heavy-atom numbering and the six central rotatable bonds. The table lists the four-atom definition of each torsion, the corresponding central rotating bond, and a plain-language structural interpretation. Each torsion is defined by four atoms; the middle two atoms define the central rotating bond used to describe conformational variation.

Individual structural interpretation of T1–T6

Each key torsion controls a chemically interpretable component of side-chain orientation or internal rotation. These panels illustrate the structural meaning of each torsion separately.

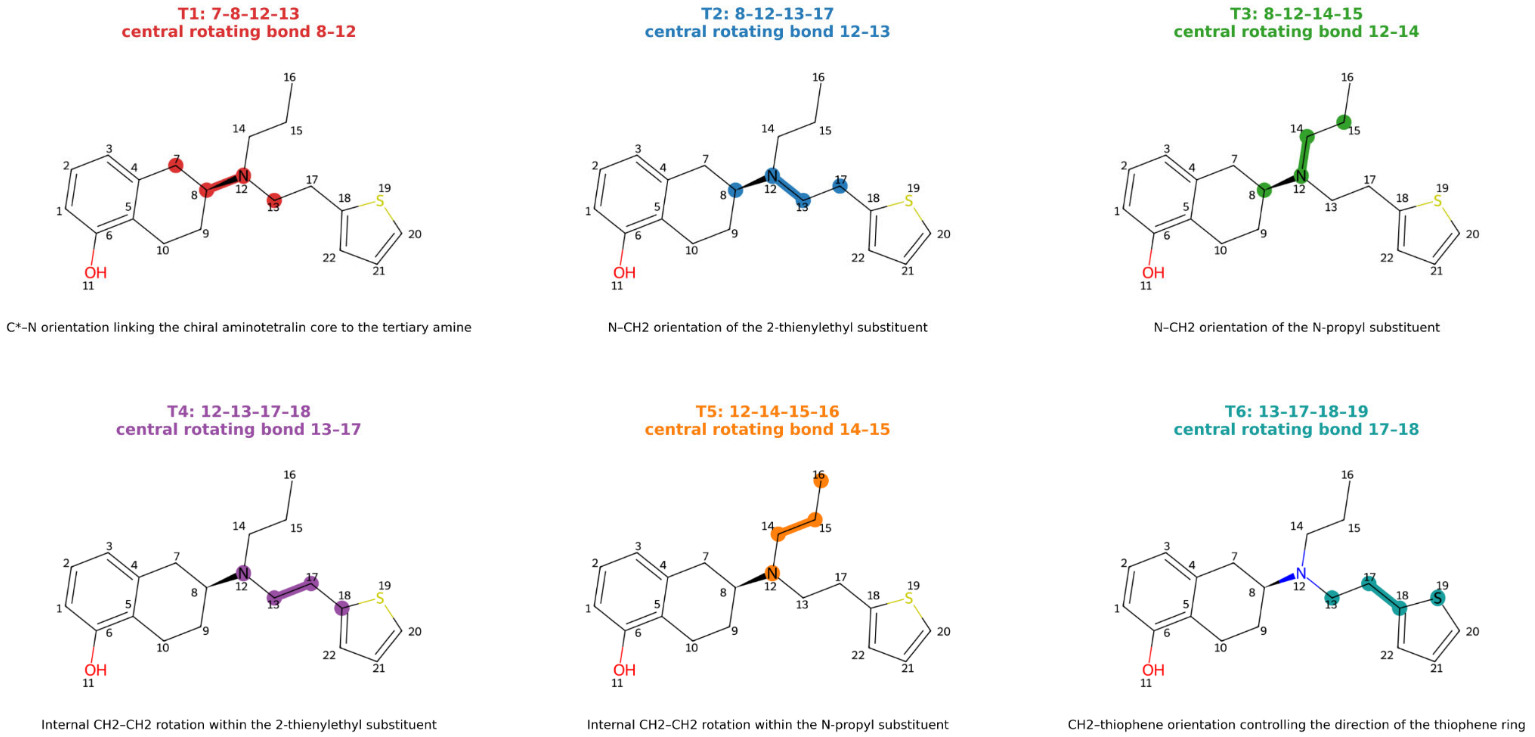

Figure S3. Individual structural interpretation of the six key torsion angles T1-T6. Each panel highlights the four atoms defining one torsion and the corresponding central rotating bond. T1 describes the C*-N orientation linking the chiral aminotetralin core to the tertiary amine; T2 and T3 describe N-CH_2 orientations of the 2-thienylethyl and N-propyl substituents, respectively; T4 and T5 describe internal CH_2-CH_2 rotations within the two substituents; and T6 describes the CH_2-thiophene orientation controlling the direction of the thiophene ring.

Table S2. Definitions and relative importance of the six key torsions used for torsion-based descriptor construction.

Torsion	Atoms (1-based)	Importance (%)
T1	7-8-12-13	10.471
T2	8-12-13-17	9.932
T3	8-12-14-15	13.957
T4	12-13-17-18	11.602
T5	12-14-15-16	8.439
T6	13-17-18-19	9.453

Atoms are numbered according to the coordinate files used for the PCA-Tor analysis. Torsion angles were encoded as sine and cosine components before descriptor standardization.

Torsional descriptor diagnostics

The torsional diagnostic plots summarize the torsion-PCA projection and the relative contribution of the six key torsions used in the PCA-Tor descriptor space.

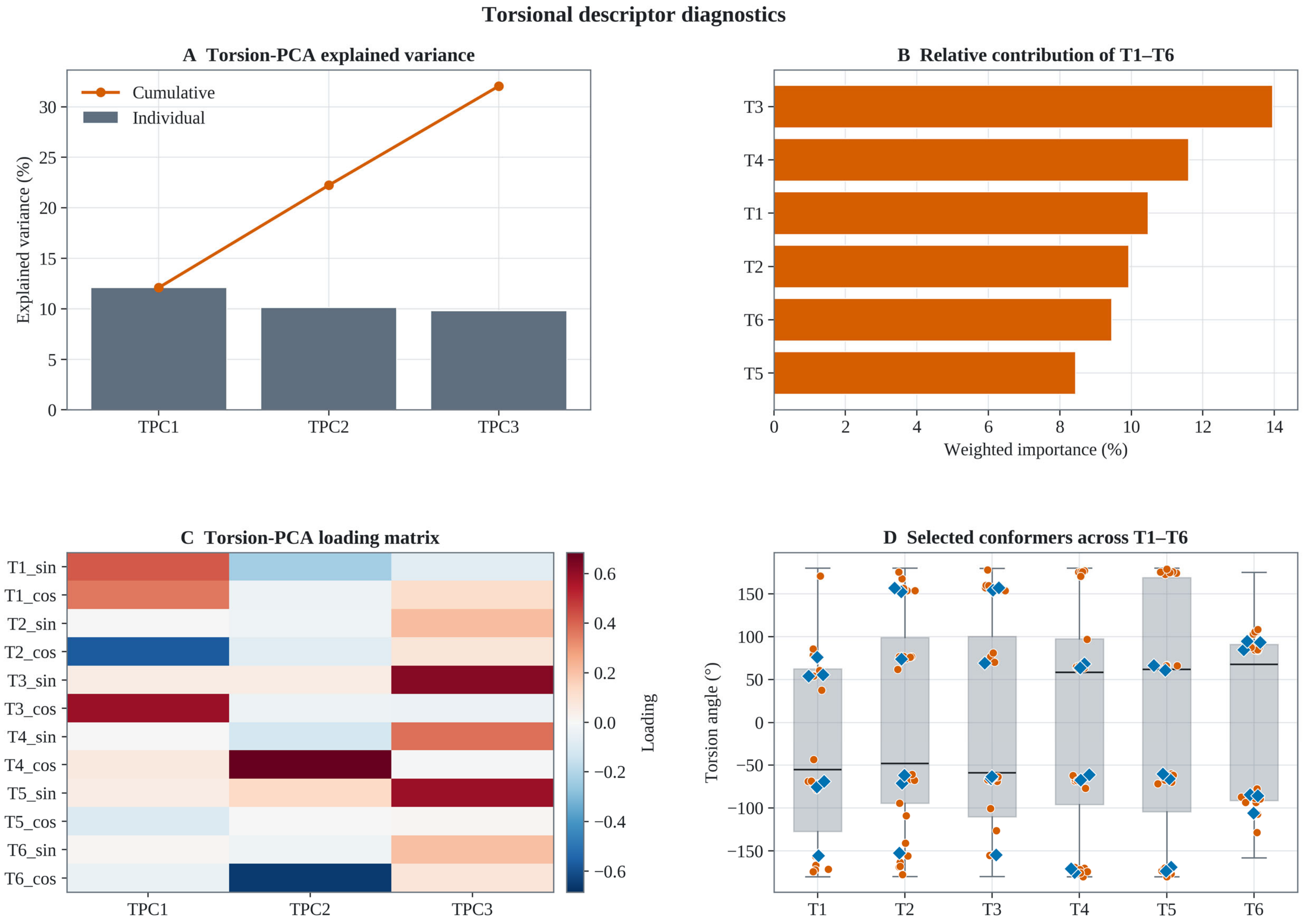

Figure S4. Torsional descriptor diagnostics. (A) Explained variance ratios of TPC1-TPC3. (B) Relative importance ranking of the six key torsions T1‑T6. (C) Torsion-PCA loading heatmap. (D) Distribution of selected conformers across the six torsion-angle coordinates. Torsions were encoded using sine and cosine components before standardization to avoid artificial discontinuities at the -180°/180° boundary.

Table S3. Explained variance ratios of the coordinate–PCA components retained in the PCA-Tor descriptor space. PCA1-PCA9 were retained as coordinate–PCA descriptors because these components cumulatively accounted for 90.756% of the total coordinate variance. Only PCA1-PCA3 were shown in the main-text three-dimensional visualization

PC	Var (%)	Cum. var (%)	Retained
PCA1	24.079	24.079	Yes
PCA2	22.695	46.774	Yes
PCA3	16.475	63.249	Yes
PCA4	9.257	72.506	Yes
PCA5	4.564	77.070	Yes
PCA6	4.272	81.342	Yes
PCA7	3.862	85.204	Yes
PCA8	3.181	88.385	Yes
PCA9	2.371	90.756	Yes

Table S4 Gaussian input validation summary for the selected conformers of (*S*)-**1**.

Input name	MOE conformer ID	Rerun/rescue	Atoms_total	Explicit_H_count	Connectivity_rows	Link1_count	Opt/freq	ECD	ORD	Validated
pair6_24_conf_01	1	No	47	25	47	2	Yes	Yes	Yes	Yes
pair6_24_conf_02	2	No	47	25	47	2	Yes	Yes	Yes	Yes
pair6_24_conf_03	9	No	47	25	47	2	Yes	Yes	Yes	Yes
pair6_24_conf_04	10	No	47	25	47	2	Yes	Yes	Yes	Yes
pair6_24_conf_05	14	No	47	25	47	2	Yes	Yes	Yes	Yes
pair6_24_conf_06	16	Yes	47	25	47	2	Yes	Yes	Yes	Yes
pair6_24_conf_07	4	No	47	25	47	2	Yes	Yes	Yes	Yes
pair6_24_conf_08	6	No	47	25	47	2	Yes	Yes	Yes	Yes
pair6_24_conf_09	12	No	47	25	47	2	Yes	Yes	Yes	Yes
pair6_24_conf_10	13	No	47	25	47	2	Yes	Yes	Yes	Yes
pair6_24_conf_11	15	No	47	25	47	2	Yes	Yes	Yes	Yes

Input name	MOE conformer ID	Rerun/rescue	Atoms_total	Explicit_H_count	Connectivity_rows	Link1_count	Opt/freq	ECD	ORD	Validated
pair6_24_conf_12	17	No	47	25	47	2	Yes	Yes	Yes	Yes
pair6_24_conf_13	21	No	47	25	47	2	Yes	Yes	Yes	Yes
pair6_24_conf_14	24	No	47	25	47	2	Yes	Yes	Yes	Yes
pair6_24_conf_15	25	No	47	25	47	2	Yes	Yes	Yes	Yes
pair6_24_conf_16	28	No	47	25	47	2	Yes	Yes	Yes	Yes
pair6_24_conf_17	30	Yes	47	25	47	2	Yes	Yes	Yes	Yes
pair6_24_conf_18	31	No	47	25	47	2	Yes	Yes	Yes	Yes
pair6_24_conf_19	34	No	47	25	47	2	Yes	Yes	Yes	Yes
pair6_24_conf_20	58	Yes	47	25	47	2	Yes	Yes	Yes	Yes
pair6_24_conf_21	84	Yes	47	25	47	2	Yes	Yes	Yes	Yes
pair6_24_conf_22	108	Yes	47	25	47	2	Yes	Yes	Yes	Yes
pair6_24_conf_23	127	Yes	47	25	47	2	Yes	Yes	Yes	Yes
pair6_24_conf_24	139	Yes	47	25	47	2	Yes	Yes	Yes	Yes
pair6_24_conf_25	156	Yes	47	25	47	2	Yes	Yes	Yes	Yes
pair6_24_conf_26	158	Yes	47	25	47	2	Yes	Yes	Yes	Yes
pair6_24_conf_27	200	Yes	47	25	47	2	Yes	Yes	Yes	Yes
pair6_24_conf_28	339	Yes	47	25	47	2	Yes	Yes	Yes	Yes
pair6_24_conf_29	360	Yes	47	25	47	2	Yes	Yes	Yes	Yes
pair6_24_conf_30	625	Yes	47	25	47	2	Yes	Yes	Yes	Yes

All selected Gaussian inputs contained the expected atom counts, connectivity section, Link1 structure, and calculation blocks. Validation was performed before quantum-chemical submission to support computational traceability.

Coordinate–PCA descriptor diagnostics

The diagnostic plots summarize how much coordinate variance was retained by PCA1-PCA9 and how the selected conformers are distributed along the retained coordinate–PCA axes.

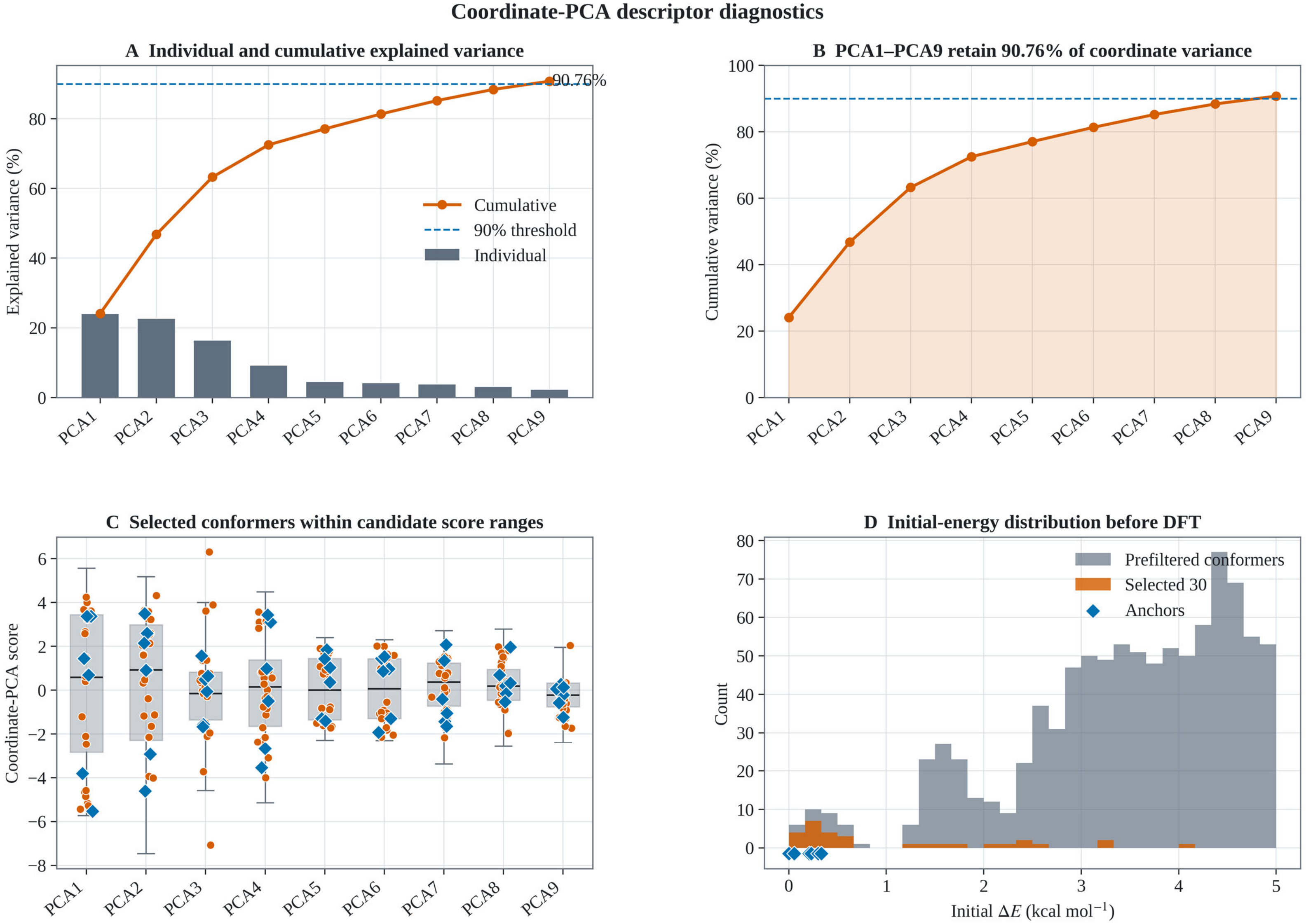

Figure S5. Coordinate–PCA descriptor diagnostics. (A) Individual explained variance ratios of PCA1-PCA9. (B) Cumulative explained variance of the retained coordinate–PCA components. PCA1-PCA9 accounted for 90.756% of the total coordinate variance. (C) Distribution of the selected conformers along PCA1-PCA9. (D) Initial relative-energy distribution of the prefiltered and selected conformers. These plots provide supporting information for retaining PCA1-PCA9 in the PCA-Tor descriptor space.

Pairwise projections of coordinate–PCA descriptors

Because PCA1-PCA9 were used as coordinate descriptors during conformer selection, all two-component projections among the retained coordinate–PCA components are shown below.

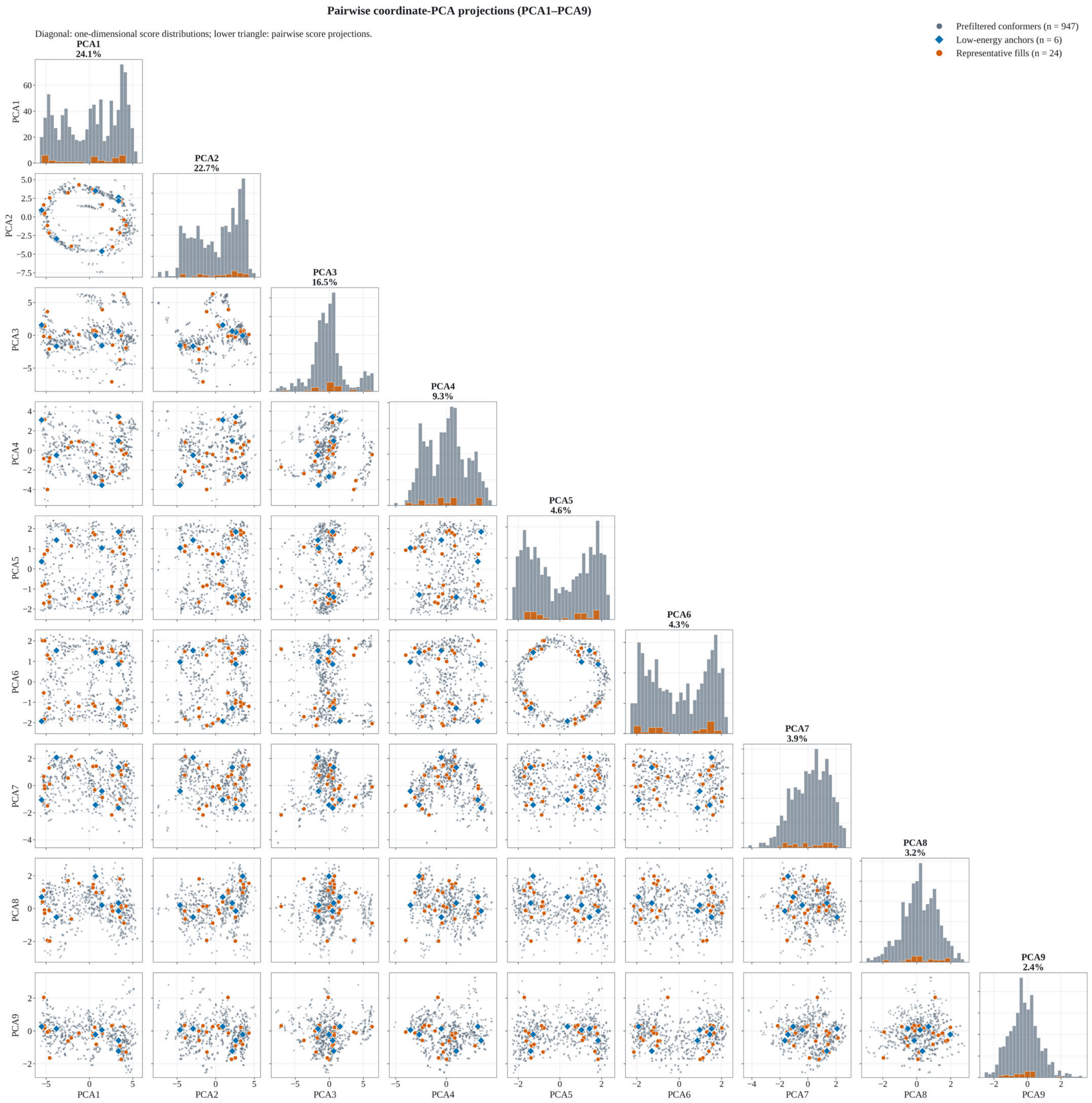

Figure S6. Pairwise coordinate–PCA projections of PCA1-PCA9. The off-diagonal panels show two-dimensional projections for each pair of retained coordinate–PCA components, whereas the diagonal panels show the corresponding one-dimensional distributions. Grey-blue points represent the prefiltered conformer pool, blue diamonds denote paired anchors, and amber circles denote additional representative conformers. This figure supports the use of PCA1-PCA9 in descriptor construction, while the main-text visualization displays only PCA1-PCA3.

Reproducible implementation of the 6+24 pair-aware PCA-Tor selection

The supplied runner separates two distinct tasks: (i) locked mode validates and exports the frozen 30-conformer manuscript set, and (ii) consensus mode re-runs a stochastic, ECD-blind sensitivity analysis. Locked mode does not re-derive the 24 representative conformers from K-means; it verifies that the archived identifiers are present in the prefiltered feature table and satisfy the predefined energy and clash criteria.

The feature table contains 1758 MOE conformers. The prefilter retains structures with initial dE ≤ 5.0 kcal mol^{-1} and Clash_count = 0, yielding 947 candidates. The selection descriptor contains PCA1-PCA9 and sine/cosine encodings of T1-T6, giving 21 standardized dimensions. Experimental ECD, calculated single-conformer ECD, ORD, and spectral-agreement metrics are not read by the runner.

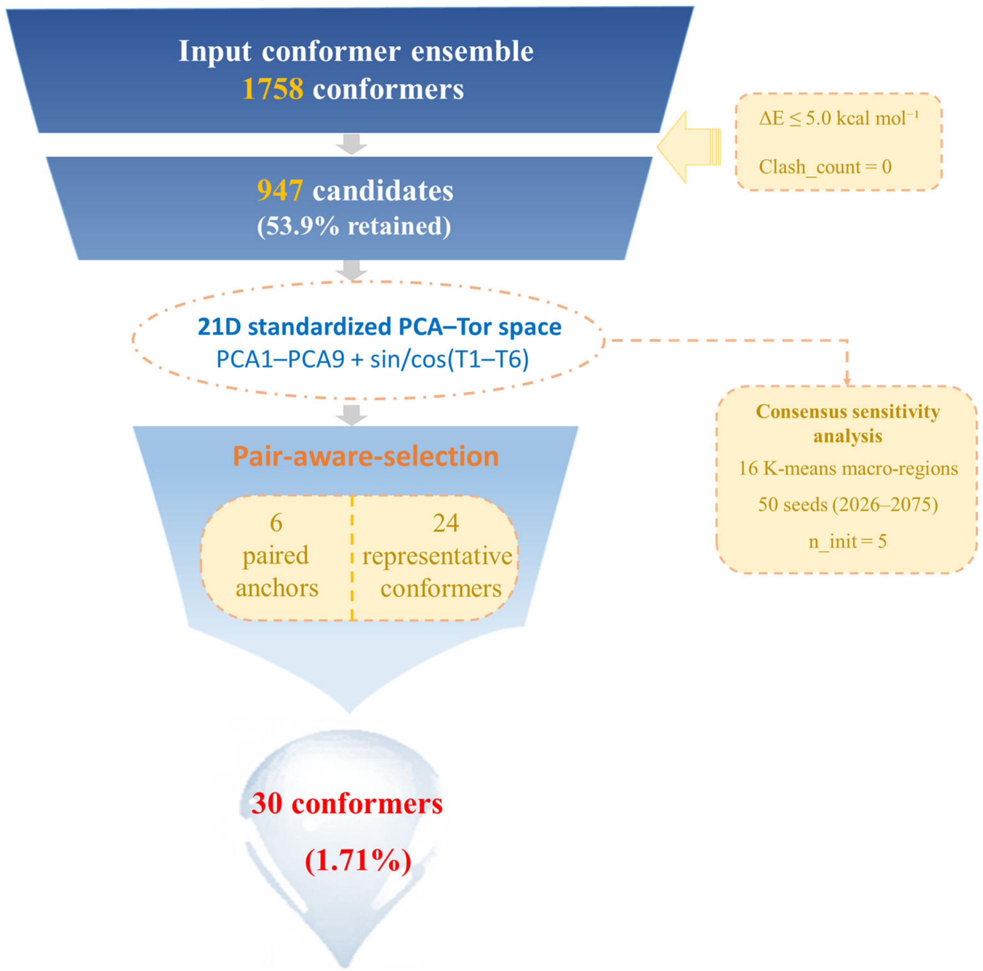

Figure S7. Reproducibility logic of the 6+24 pair-aware PCA-Tor runner. K-means macro-regions are operational sampling regions and must not be interpreted as thermodynamic conformer families. The paired anchors protect low-initial-energy, structurally complementary alternatives but are not asserted to be rigorously degenerate states.

PCA-Tor visualization of the selected rotigotine conformers

The following projections provide a low-dimensional overview of how the selected conformers cover the prefiltered rotigotine conformer pool in coordinate- and torsion-based descriptor spaces.

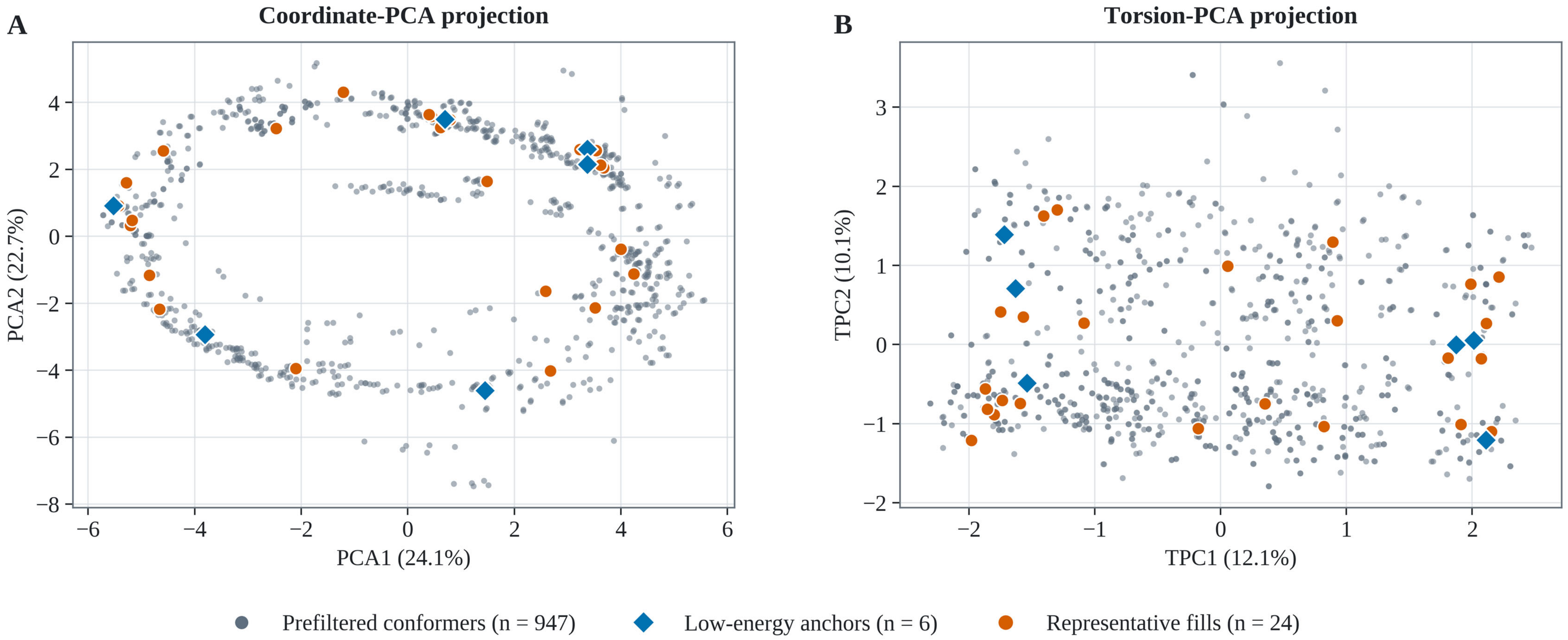

Figure S8. Two-dimensional PCA-Tor projections of the selected rotigotine conformers. (A) Coordinate–PCA projection in the PCA1-PCA2 plane. (B) Torsion-PCA projection in the TPC1-TPC2 plane. Grey-blue points represent the prefiltered rotigotine conformer pool; blue diamonds indicate paired low-initial-energy anchors; amber circles indicate additional representative conformers. These two-dimensional projections are provided for visual inspection only; conformer selection was performed in the full standardized PCA-Tor descriptor space.

Table S5. Final ACN 30-conformer Boltzmann populations at 298.15 K.

Input	MOE ID	Gibbs (Eh)	Delta G (kcal mol^{-1})	Population (%)
pair6_24_conf_01	1	-1267.040670000	0.540	6.2687
pair6_24_conf_02	2	-1267.041013000	0.325	9.0146
pair6_24_conf_03	9	-1267.041335000	0.123	12.6782
pair6_24_conf_04	10	-1267.039662000	1.173	2.1554
pair6_24_conf_05	14	-1267.038866000	1.672	0.9277
pair6_24_conf_06	16	-1267.038634000	1.818	0.7256
pair6_24_conf_07	4	-1267.040374000	0.726	4.5817
pair6_24_conf_08	6	-1267.041531000	0.000	15.6031
pair6_24_conf_09	12	-1267.039241000	1.437	1.3800
pair6_24_conf_10	13	-1267.038759000	1.739	0.8283
pair6_24_conf_11	15	-1267.040353000	0.739	4.4809
pair6_24_conf_12	17	-1267.040079000	0.911	3.3523
pair6_24_conf_13	21	-1267.039882000	1.035	2.7210
pair6_24_conf_14	24	-1267.039945000	0.995	2.9087
pair6_24_conf_15	25	-1267.039885000	1.033	2.7296
pair6_24_conf_16	28	-1267.039319000	1.388	1.4989
pair6_24_conf_17	30	-1267.040916000	0.386	8.1345
pair6_24_conf_18	31	-1267.039093000	1.530	1.1798
pair6_24_conf_19	34	-1267.040415000	0.700	4.7851
pair6_24_conf_20	58	-1267.037198000	2.719	0.1586
pair6_24_conf_21	84	-1267.037608000	2.462	0.2448
pair6_24_conf_22	108	-1267.036884000	2.916	0.1137
pair6_24_conf_23	127	-1267.039522000	1.261	1.8584
pair6_24_conf_24	139	-1267.037593000	2.471	0.2409
pair6_24_conf_25	156	-1267.034995000	4.101	0.0154
pair6_24_conf_26	158	-1267.035705000	3.656	0.0326
pair6_24_conf_27	200	-1267.039729000	1.131	2.3139
pair6_24_conf_28	339	-1267.035996000	3.473	0.0444
pair6_24_conf_29	360	-1267.040990000	0.339	8.7977
pair6_24_conf_30	625	-1267.037531000	2.510	0.2256

Table S6. Final MeOH 30-conformer Boltzmann populations at 298.15 K.

Input	MOE ID	Gibbs (Eh)	Delta G (kcal mol^{-1})	Population (%)
pair6_24_conf_01	1	-1267.039386000	0.956	3.7437
pair6_24_conf_02	2	-1267.040429000	0.301	11.2993
pair6_24_conf_03	9	-1267.040012000	0.563	7.2652
pair6_24_conf_04	10	-1267.038828000	1.306	2.0732
pair6_24_conf_05	14	-1267.038807000	1.319	2.0276
pair6_24_conf_06	16	-1267.038652000	1.416	1.7206
pair6_24_conf_07	4	-1267.038994000	1.202	2.4717
pair6_24_conf_08	6	-1267.040909000	0.000	18.7861
pair6_24_conf_09	12	-1267.039447000	0.917	3.9936
pair6_24_conf_10	13	-1267.037787000	1.959	0.6884
pair6_24_conf_11	15	-1267.039877000	0.648	6.2972
pair6_24_conf_12	17	-1267.039217000	1.062	3.1302
pair6_24_conf_13	21	-1267.038852000	1.291	2.1266
pair6_24_conf_14	24	-1267.039188000	1.080	3.0355
pair6_24_conf_15	25	-1267.039360000	0.972	3.6421
pair6_24_conf_16	28	-1267.038395000	1.578	1.3106
pair6_24_conf_17	30	-1267.039635000	0.799	4.8735
pair6_24_conf_18	31	-1267.038124000	1.748	0.9836
pair6_24_conf_19	34	-1267.040223000	0.430	9.0844
pair6_24_conf_20	58	-1267.037440000	2.177	0.4767
pair6_24_conf_21	84	-1267.037332000	2.245	0.4251
pair6_24_conf_22	108	-1267.037091000	2.396	0.3294
pair6_24_conf_23	127	-1267.038767000	1.344	1.9435
pair6_24_conf_24	139	-1267.037390000	2.208	0.4521
pair6_24_conf_25	156	-1267.034176000	4.225	0.0150
pair6_24_conf_26	158	-1267.035137000	3.622	0.0416
pair6_24_conf_27	200	-1267.038464000	1.534	1.4100
pair6_24_conf_28	339	-1267.035320000	3.507	0.0505
pair6_24_conf_29	360	-1267.039856000	0.661	6.1587
pair6_24_conf_30	625	-1267.036312000	2.885	0.1443

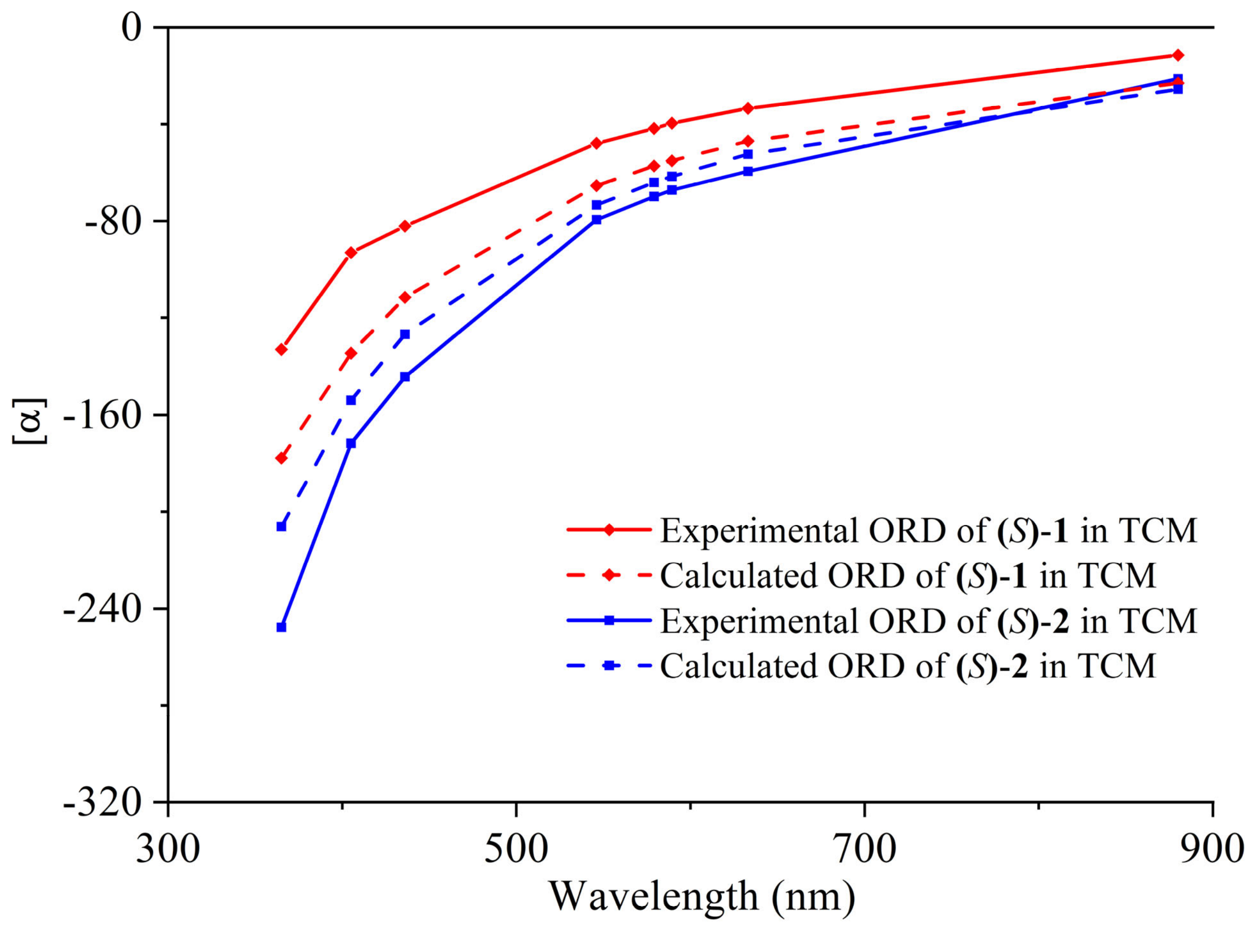

Figure S9. Comparison of the calculated (dashed line) and experimental (solid line) ORD curves of (*S*)-**1** (red) and (*S*)-**2** (blue) in chloroform. (*S*)-**1** calculated at the SMD/solvent/Cam-B3LYP/Aug-cc-pVDZ//ωB97X-D/TZVP level; (*S*)-**2** calculated at the SMD/solvent/ωB97X-D/Aug-cc-pVDZ//ωB97X-D/TZVP level.

Table S7. Cartesian coordinates of main conformers (Boltzmann population >0.5%) of (*S*)-**1** calculated at the B3LYP/6-31G(d,p) level in acetonitrile (sorted in descending order of content).

Conformers	Cartesian coordinates				Conformers	Cartesian coordinates			
(*S*)-**1C1**	C	5.21216700	-1.41748300	0.06670900	(*S*)-**1C12**	C	5.66931000	-0.39988900	0.30809100
	C	4.35970900	-2.27324300	0.76205200		C	5.38816900	0.65849600	1.16996200
	C	3.02869500	-1.91892100	0.96874800		C	4.08878600	1.14988200	1.27376300
	C	2.53187500	-0.69668600	0.49405100		C	3.05265700	0.59856200	0.50690700
	C	3.38101300	0.18270100	-0.20021300		C	3.32058400	-0.46446800	-0.37290800
	C	4.71915000	-0.20392900	-0.41629100		C	4.63788300	-0.95921200	-0.44878200
	C	1.08001100	-0.34433300	0.74992600		C	1.65486700	1.17127600	0.62765800
	C	0.55560200	0.76973100	-0.15967200		C	0.56347800	0.19812400	0.14956400
	C	1.54638500	1.94356400	-0.14837900		C	0.93075700	-0.30398200	-1.24871900
	C	2.88899400	1.50966800	-0.74408300		C	2.23216100	-1.11229400	-1.20668400
	O	5.59950900	0.58331500	-1.10633900		O	4.97407900	-2.00659700	-1.26121600
	N	-0.83547900	1.10980700	0.17097500		N	-0.80356700	0.73042600	0.20972300
	C	-1.57623200	1.64193000	-0.96926800		C	-1.36854900	0.69755000	1.55663800
	C	-1.01320900	1.90689100	1.38937600		C	-1.02711200	2.00099000	-0.48418300
	C	-2.34633600	1.62097500	2.09077800		C	-2.44215800	2.10600600	-1.05980400
	C	-2.47987800	0.17432600	2.56507100		C	-2.69597000	3.45700500	-1.72907300
	C	-2.04837800	0.54264500	-1.94085900		C	-2.02537000	-0.65465100	1.89942300
	C	-2.90542500	-0.49712800	-1.28694100		C	-3.23654900	-0.95337900	1.06771000
	S	-4.51841000	-0.12008200	-0.71499200		S	-3.11979400	-1.79948200	-0.45736300
	C	-4.73081300	-1.72081500	-0.06665700		C	-4.82887800	-1.66085300	-0.74157900
	C	-3.62568100	-2.49405500	-0.28556000		C	-5.44642700	-0.98956600	0.27784200
	C	-2.58694100	-1.79368400	-0.97885400		C	-4.53839100	-0.59105100	1.30836400
	H	6.25070100	-1.67419900	-0.11596500		H	6.67094200	-0.80733300	0.21566400
	H	4.73644200	-3.22141700	1.13461300		H	6.18554700	1.09292100	1.76599400
	H	2.36074500	-2.59084100	1.50069800		H	3.86657800	1.96840100	1.95286100
	H	0.45634200	-1.23634900	0.62906300		H	1.47656900	1.47125000	1.66570600
	H	0.97094500	-0.03789400	1.79859100		H	1.59559600	2.08873200	0.02731100
	H	0.53798400	0.37041500	-1.18059800		H	0.57567900	-0.67325300	0.81653800
	H	1.15008600	2.79095300	-0.71709600		H	0.11994600	-0.92175600	-1.64611700
	H	1.69655500	2.28358700	0.88286600		H	1.05437300	0.54791600	-1.92771300
	H	3.63287000	2.29599200	-0.55439200		H	2.58574900	-1.26602500	-2.23557400
	H	2.79293100	1.43985200	-1.83775800		H	2.02081200	-2.11442700	-0.80510300
	H	5.14916500	1.38087400	-1.42235900		H	4.18499500	-2.34276400	-1.71180000
	H	-2.45719200	2.16677500	-0.59061600		H	-0.61095900	0.92040900	2.32567900
	H	-0.99244700	2.38628500	-1.53796400		H	-2.13159600	1.47600300	1.63797200
	H	-0.20409800	1.66095900	2.08342400		H	-0.83452300	2.86908800	0.17182900
	H	-0.92935100	2.98885600	1.18639100		H	-0.31440500	2.07905700	-1.31022400
	H	-2.43147200	2.30613300	2.94419900		H	-2.58491600	1.29114000	-1.77907400
	H	-3.17850500	1.86322600	1.41923400		H	-3.17880200	1.94388700	-0.26370700
	H	-2.38578800	-0.51877700	1.72586600		H	-1.98865200	3.63423300	-2.54805000
	H	-1.69970200	-0.07407800	3.29507700		H	-3.70718200	3.51175800	-2.14567400
	H	-3.45053100	-0.00020200	3.04161500		H	-2.58580600	4.28089600	-1.01413700
	H	-2.58163800	1.02696500	-2.76787600		H	-1.28400500	-1.45490800	1.80203500
	H	-1.18527500	0.03396000	-2.38203900		H	-2.32549400	-0.63037500	2.95329400
	H	-5.65770000	-1.98827400	0.42278400		H	-5.26793700	-2.07818800	-1.63794300
	H	-3.54887000	-3.52704900	0.03520900		H	-6.51223600	-0.79043100	0.30063300
	H	-1.62960200	-2.23253500	-1.23880600		H	-4.83673000	-0.05598600	2.20393100
(*S*)-**1C2**	C	-4.57153600	-1.50989400	0.58117900	(*S*)-**1C13**	C	-5.46480500	-1.18653100	-0.73912900
	C	-3.53151500	-2.31380600	1.04489300		C	-5.20000200	-0.18816300	-1.67496800
	C	-2.20954800	-1.90050700	0.89847700		C	-4.00624100	0.52712600	-1.61662300
	C	-1.90727700	-0.68189300	0.27440200		C	-3.06363300	0.26435400	-0.61241200
	C	-2.94290300	0.13444200	-0.21188200		C	-3.32051800	-0.73114100	0.34635700
	C	-4.27435400	-0.29273900	-0.03497400		C	-4.52491300	-1.45796100	0.25687000
	C	-0.45621100	-0.27502100	0.12831600		C	-1.78052600	1.07133400	-0.57814700
	C	-0.26924800	1.21822400	-0.15207100		C	-0.67693200	0.41741600	0.25642800
	C	-1.19959500	1.63600200	-1.30149800		C	-1.24921200	0.01120900	1.62233900
	C	-2.66551900	1.46258700	-0.88948300		C	-2.33076600	-1.05855300	1.44697500
	O	-5.33880500	0.45797400	-0.45235100		O	-4.83497400	-2.45972800	1.13541700
	N	1.15190400	1.54349700	-0.32885900		N	0.52820200	1.26182100	0.30106200
	C	1.76749100	1.04065200	-1.55666800		C	1.74593800	0.48053900	0.51675100
	C	1.48520100	2.94942500	-0.08732500		C	0.43825000	2.42836500	1.18859300
	C	1.33864700	3.39343800	1.37211000		C	1.29683000	3.60005900	0.69907800
	C	2.15709600	2.55390800	2.35276400		C	0.84864700	4.14538500	-0.65663200
	C	3.15383900	0.41406600	-1.29107700		C	2.28389700	-0.11626500	-0.79691000
	C	3.07105000	-0.82969000	-0.46035700		C	3.49088500	-0.98050200	-0.59293500
	S	2.57942900	-2.35565800	-1.17197500		S	5.03554400	-0.29159300	-0.12494200
	C	2.57973800	-3.15028300	0.37629800		C	5.78363400	-1.86173900	-0.05447900
	C	2.94546600	-2.28966100	1.37295700		C	4.89337500	-2.84904300	-0.36950200

	C	3.22211400	-0.96900200	0.89436300		C	3.58875800	-2.34495700	-0.67301900
	H	-5.61032100	-1.80407100	0.69181100		H	-6.38242800	-1.76520400	-0.76944500
	H	-3.75782400	-3.25961100	1.52873400		H	-5.92510400	0.02278100	-2.45565200
	H	-1.39674800	-2.51972000	1.26838400		H	-3.79436200	1.29713100	-2.35333600
	H	0.09747900	-0.55282200	1.03060300		H	-1.41902700	1.23216000	-1.59934300
	H	-0.00947400	-0.85743800	-0.68672400		H	-2.00015400	2.06766100	-0.17226100
	H	-0.59682400	1.75725900	0.74350100		H	-0.38286300	-0.50382000	-0.26083000
	H	-1.02101100	2.67776400	-1.58616400		H	-0.45887500	-0.37118200	2.27619400
	H	-0.99140700	1.01658500	-2.18200900		H	-1.68153800	0.89143800	2.11198700
	H	-3.29903800	1.57037900	-1.78097400		H	-2.85900400	-1.18975000	2.40142000
	H	-2.94993800	2.28185000	-0.21283100		H	-1.85196200	-2.02474400	1.22891800
	H	-5.02318200	1.28150200	-0.85349000		H	-4.09825400	-2.59856000	1.74941400
	H	1.12804300	0.27919700	-2.00887300		H	2.51177000	1.13703200	0.93814200
	H	1.87739800	1.83656600	-2.30984000		H	1.59594900	-0.33072300	1.24868300
	H	2.53414100	3.08141000	-0.37555800		H	-0.60397600	2.75918900	1.22108900
	H	0.89953200	3.62815700	-0.73486500		H	0.71705400	2.17360600	2.22526100
	H	1.65629400	4.44246000	1.42450500		H	1.25095900	4.39337400	1.45603200
	H	0.28353700	3.38601800	1.67052500		H	2.34855100	3.29125800	0.64421700
	H	2.06987300	2.94106100	3.37369300		H	-0.17996900	4.52255700	-0.60843500
	H	1.82396500	1.51275700	2.35513500		H	1.49091900	4.96801600	-0.98880400
	H	3.22000300	2.56060800	2.08262900		H	0.87899600	3.36062600	-1.41818000
	H	3.63520500	0.20714500	-2.25425300		H	1.51059700	-0.73092300	-1.26888500
	H	3.78414800	1.14085700	-0.76794500		H	2.50141400	0.70810400	-1.48551700
	H	2.33167500	-4.20094400	0.44679500		H	6.82875700	-1.95235500	0.21008600
	H	3.01843400	-2.58283600	2.41444600		H	5.15417200	-3.90141900	-0.38833700
	H	3.51466600	-0.14335700	1.53263500		H	2.74604900	-2.97129100	-0.94592600
(*S*)-**1C3**	C	5.23746500	-0.77018800	-0.00761900	(*S*)-**1C14**	C	5.80477900	-0.51266000	-0.58045100
	C	5.19074300	0.31440800	-0.88158400		C	5.16289300	-1.45375700	-1.38342300
	C	3.97231700	0.91935600	-1.18152800		C	3.77517800	-1.56948000	-1.35319000
	C	2.77872800	0.43901100	-0.62414000		C	3.00790000	-0.75396900	-0.50930400
	C	2.80749300	-0.65989500	0.25192600		C	3.64035700	0.19310800	0.31483900
	C	4.05272100	-1.24363600	0.55920700		C	5.04375200	0.30793100	0.25411800
	C	1.46879700	1.11073200	-0.98624600		C	1.50086000	-0.91825500	-0.49760600
	C	0.34482100	0.81801100	0.02228200		C	0.76490200	0.28187600	0.10282700
	C	0.26102700	-0.69459400	0.24524100		C	1.40946000	0.64702200	1.44899200
	C	1.54898800	-1.20768600	0.89617400		C	2.85525100	1.10606500	1.23609600
	O	4.16477700	-2.29758800	1.42357500		O	5.72906700	1.22411800	1.00350200
	N	-0.95911800	1.40057600	-0.31617900		N	-0.68835300	0.05331400	0.13087400
	C	-1.53039200	0.97621000	-1.59317800		C	-1.14733200	-0.90697600	1.13684300
	C	-1.04272500	2.85254000	-0.13953300		C	-1.45812700	1.30357000	0.12619500
	C	-0.90554100	3.32170500	1.31252500		C	-1.57765600	1.93521300	-1.26199500
	C	-1.90899900	2.66839900	2.26276600		C	-2.53823800	3.12509600	-1.26277600
	C	-3.04388100	0.69147700	-1.48180300		C	-2.20093900	-1.89136100	0.58284300
	C	-3.35229000	-0.46499800	-0.58128700		C	-3.46114600	-1.24588200	0.09374800
	S	-3.12670300	-2.11722400	-1.12337300		S	-4.67437300	-0.64974000	1.21222800
	C	-3.53976200	-2.75408900	0.44242800		C	-5.62180200	0.02797900	-0.08243700
	C	-3.83724200	-1.75063100	1.32136500		C	-5.04043500	-0.20663300	-1.29678400
	C	-3.72784000	-0.44857400	0.73647300		C	-3.81137700	-0.93331800	-1.19344800
	H	6.17471900	-1.25242900	0.25093500		H	6.88367200	-0.39581800	-0.59270400
	H	6.11010700	0.69141600	-1.32026900		H	5.74967100	-2.09061800	-2.03907200
	H	3.93669200	1.77239700	-1.85358200		H	3.27358100	-2.29512400	-1.98754200
	H	1.63325300	2.18954800	-1.07693200		H	1.13996400	-1.09516700	-1.51645000
	H	1.15282500	0.76319000	-1.97925700		H	1.24953500	-1.82102500	0.07540100
	H	0.63540500	1.26059500	0.98139400		H	0.92182200	1.13018900	-0.57340000
	H	-0.60038700	-0.93319300	0.87563700		H	0.84338600	1.43984300	1.94799400
	H	0.11217300	-1.20705900	-0.71170400		H	1.39817600	-0.22794600	2.10930700
	H	1.55227600	-2.30581400	0.85668700		H	3.35375100	1.17663700	2.21299400
	H	1.54429700	-0.94170000	1.96367300		H	2.85332600	2.12499100	0.82162200
	H	3.29579900	-2.51324800	1.79361100		H	5.10688000	1.75320000	1.52444000
	H	-1.37181400	1.72882400	-2.38238400		H	-0.30284900	-1.50939800	1.47916600
	H	-1.03496300	0.06468300	-1.93402600		H	-1.54971800	-0.40332500	2.02995800
	H	-0.30644400	3.38833600	-0.76651900		H	-2.46493200	1.07867400	0.48197800
	H	-2.03076800	3.15957500	-0.50080100		H	-1.04054900	2.04271900	0.83351800
	H	0.11493400	3.15044000	1.67543000		H	-0.59210500	2.25530400	-1.62172300
	H	-1.04431700	4.41009900	1.31536000		H	-1.93241800	1.16700300	-1.95834700
	H	-1.76111000	1.58633300	2.31221200		H	-2.21269600	3.90220900	-0.56116700
	H	-1.81380000	3.07167400	3.27673100		H	-2.60844000	3.58213300	-2.25534400
	H	-2.93790400	2.84605600	1.92760900		H	-3.54633300	2.81122400	-0.96685500
	H	-3.55259200	1.57842300	-1.08991800		H	-1.75305300	-2.43424500	-0.25579400
	H	-3.43975000	0.51639500	-2.48921900		H	-2.42494300	-2.62635700	1.36574900
	H	-3.55163200	-3.82308200	0.60769200		H	-6.55342300	0.53318800	0.13486500
	H	-4.12705500	-1.92848500	2.35113500		H	-5.47142700	0.12221000	-2.23586500
	H	-3.91366600	0.47394400	1.27432700		H	-3.19463700	-1.20422000	-2.04303300

(*S*)-**1C4**	C	-0.64875700	-3.63601900	-0.94773700	(*S*)-**1C15**	C	5.08374400	-1.45593300	0.21877700
	C	0.72572300	-3.80689700	-1.10166300		C	4.20482300	-2.22695500	0.97739900
	C	1.61426200	-2.98142100	-0.41329400		C	2.88460800	-1.81766900	1.14889800
	C	1.13856100	-1.99343800	0.45827700		C	2.42569300	-0.62376700	0.57453900
	C	-0.24394500	-1.82223400	0.63960700		C	3.30187800	0.17099900	-0.18515800
	C	-1.12474700	-2.64002500	-0.09183600		C	4.62834200	-0.27136100	-0.36314500
	C	2.11482400	-1.11374900	1.20985900		C	0.98411100	-0.20997500	0.79318400
	C	1.52012300	0.26008300	1.55562200		C	0.49271400	0.82970700	-0.21698200
	C	0.25619400	-0.02808300	2.38591200		C	1.51205800	1.97623900	-0.29677600
	C	-0.80649600	-0.76410800	1.56295600		C	2.85006400	1.46305400	-0.83775300
	O	-2.48417000	-2.51102200	0.01699200		O	5.53377300	0.43026000	-1.11088800
	N	1.19985900	1.03545300	0.34224100		N	-0.89242700	1.23168500	0.06422900
	C	0.68323200	2.37614000	0.60981800		C	-1.60296000	1.68612400	-1.12823900
	C	2.23457100	1.03199800	-0.69698000		C	-1.06952900	2.13024800	1.20970300
	C	3.61305000	1.59601900	-0.31522500		C	-2.41849400	1.92661200	1.91003300
	C	4.58421700	1.53690100	-1.49514500		C	-2.57592000	0.53422800	2.52027100
	C	-0.26281500	2.89316400	-0.49665700		C	-2.12550000	0.52510400	-1.99515800
	C	-1.47682400	2.03739000	-0.69323800		C	-3.07999300	-0.36382400	-1.25183700
	S	-2.88922900	2.21991700	0.32973600		S	-2.62431400	-1.97989500	-0.76679400
	C	-3.68749400	0.86983500	-0.42408000		C	-4.14062200	-2.23726700	0.04377000
	C	-2.91080700	0.32465800	-1.41036400		C	-4.94559900	-1.13626800	-0.05843500
	C	-1.65144800	0.98800900	-1.55739400		C	-4.34048600	-0.07116200	-0.79760100
	H	-1.36197100	-4.24928400	-1.48930600		H	6.11441800	-1.75745600	0.06190100
	H	1.10127400	-4.57385400	-1.77291700		H	4.55236800	-3.15264200	1.42695000
	H	2.68613400	-3.09807200	-0.54873300		H	2.19547600	-2.42404700	1.73029400
	H	2.38506100	-1.59190400	2.15984300		H	0.33765400	-1.09272700	0.75256900
	H	3.04579600	-1.03094800	0.64527800		H	0.88275800	0.19483600	1.80883200
	H	2.24535000	0.79266100	2.19551300		H	0.47376900	0.34109700	-1.19861700
	H	0.56395600	-0.64190100	3.23978100		H	1.14188400	2.78089900	-0.94023200
	H	-0.17868800	0.88221500	2.80674000		H	1.66015100	2.39993000	0.70336100
	H	-1.35437700	-0.03235900	0.96144700		H	3.61131400	2.24321000	-0.69840900
	H	-1.54243200	-1.21659900	2.24075700		H	2.76594000	1.30984400	-1.92386200
	H	-2.69983600	-1.62366800	0.34540000		H	5.10932100	1.21349500	-1.49187100
	H	0.12424300	2.35897300	1.54611300		H	-2.46205800	2.28219400	-0.81105000
	H	1.48758500	3.11856000	0.74437400		H	-0.98093100	2.34523100	-1.75855900
	H	2.35577200	0.00896000	-1.06789800		H	-0.27557200	1.92848400	1.93475700
	H	1.84934200	1.60502200	-1.54699600		H	-0.96204600	3.18991100	0.91907400
	H	3.51039700	2.63307000	0.02564700		H	-2.51354400	2.69212300	2.69094500
	H	4.02667200	1.03105800	0.52846700		H	-3.23510000	2.10943400	1.20095600
	H	5.56913700	1.93341700	-1.22698000		H	-1.81206200	0.35186400	3.28599400
	H	4.72326900	0.50513900	-1.83959600		H	-3.55694900	0.41485500	2.99247100
	H	4.21034500	2.12008800	-2.34508200		H	-2.47263100	-0.23813400	1.75463000
	H	-0.55005300	3.91839000	-0.23768900		H	-2.63049300	0.95502100	-2.86851100
	H	0.27182100	2.95275400	-1.44955100		H	-1.28633900	-0.06142100	-2.38194300
	H	-4.68351900	0.58443100	-0.11307300		H	-4.34463600	-3.18008700	0.53328100
	H	-3.22052500	-0.52372700	-2.00925600		H	-5.93870200	-1.08337900	0.37400900
	H	-0.89129200	0.68956700	-2.27029000		H	-4.82173900	0.88310500	-0.98371500
(*S*)-**1C5**	C	5.43400300	-1.17245900	0.57239800	(*S*)-**1C16**	C	4.72687100	-1.20098300	-0.33913000
	C	4.62503200	-1.74366400	1.55280600		C	3.82245500	-2.15469900	-0.80388200
	C	3.27133500	-1.42240600	1.61643300		C	2.45603200	-1.88478300	-0.79851200
	C	2.70803700	-0.51470000	0.70836500		C	1.97211800	-0.66089400	-0.31465300
	C	3.51192900	0.07883300	-0.28061500		C	2.86888500	0.30491200	0.17244900
	C	4.87474700	-0.27575200	-0.33992000		C	4.24878500	0.02157700	0.13638800
	C	1.23338800	-0.18424000	0.82076400		C	0.47952800	-0.40735300	-0.32172400
	C	0.65315800	0.44737100	-0.44691000		C	0.09732700	1.07115400	-0.17616800
	C	1.56618700	1.59426200	-0.90531100		C	0.88602200	1.66248400	1.00573500
	C	2.94645200	1.05498800	-1.29446400		C	2.38921200	1.63894400	0.70854600
	O	5.71721300	0.23483800	-1.28810700		O	5.18496600	0.92346700	0.56196100
	N	-0.76080300	0.79908400	-0.26266200		N	-1.35892300	1.22034600	-0.13412000
	C	-1.51317800	0.79179100	-1.51455000		C	-2.04045500	0.84672100	1.09671800
	C	-1.02543800	1.97169200	0.57487700		C	-1.90616500	2.39262000	-0.80640700
	C	-2.33852700	1.83954100	1.35134300		C	-1.57057200	3.77312900	-0.21535600
	C	-2.62485400	3.06316800	2.22182600		C	-2.32349000	4.89268800	-0.93603200
	C	-2.04935200	-0.61158900	-1.86842400		C	-3.28727700	-0.03312200	0.83858800
	C	-3.04354700	-1.12261500	-0.87154800		C	-2.94653800	-1.35247200	0.21934500
	S	-4.72898000	-0.64645400	-0.95075800		S	-2.35707200	-2.68513900	1.19511800
	C	-5.10432400	-1.49680000	0.52046000		C	-2.05717900	-3.66532600	-0.21143500
	C	-3.99500300	-2.10823300	1.03375400		C	-2.39923500	-3.00288000	-1.35698100
	C	-2.82314100	-1.89119200	0.24129100		C	-2.90268400	-1.68610800	-1.10884300
	H	6.48939500	-1.41299100	0.49422700		H	5.79685900	-1.38257500	-0.34336400
	H	5.05339800	-2.44687900	2.26114800		H	4.18984800	-3.10580100	-1.17845100

	H	2.63698200	-1.87597100	2.37302200		H	1.74951100	-2.62194800	-1.17051900
	H	0.66968500	-1.09001900	1.06844800		H	0.04192900	-0.80956400	-1.24052300
	H	1.09048500	0.50254500	1.66558200		H	0.01898100	-0.97116200	0.49973100
	H	0.67344800	-0.32078300	-1.23019100		H	0.42457500	1.59432600	-1.08394800
	H	1.12544300	2.12222600	-1.75715900		H	0.56961100	2.68720800	1.21419300
	H	1.67245600	2.32003200	-0.09054000		H	0.68643600	1.07025000	1.90679400
	H	3.63277900	1.90253100	-1.43147200		H	2.93638300	1.89544200	1.62660000
	H	2.87687400	0.55945900	-2.27406900		H	2.62567500	2.43144900	-0.01705200
	H	5.22630500	0.81676600	-1.88688200		H	4.74827600	1.74350400	0.83736700
	H	-2.36210400	1.47458600	-1.42188800		H	-1.35392100	0.28618100	1.73554500
	H	-0.90833400	1.15922400	-2.35951500		H	-2.35681500	1.72537000	1.67996200
	H	-0.21022100	2.07848000	1.29647400		H	-1.57523300	2.37168800	-1.85354400
	H	-1.03871000	2.90616100	-0.01413900		H	-2.99775800	2.28063100	-0.83022100
	H	-3.16800200	1.68349000	0.65122000		H	-1.81860900	3.78760900	0.85318600
	H	-2.28446300	0.93513000	1.96916500		H	-0.49141100	3.94985300	-0.29095500
	H	-1.82572800	3.22507800	2.95498000		H	-2.06681500	5.87677000	-0.52992600
	H	-2.70474200	3.97247900	1.61455600		H	-2.08606100	4.90304900	-2.00659800
	H	-3.56392400	2.94869500	2.77328700		H	-3.40824900	4.76479000	-0.84047300
	H	-2.48862900	-0.57373500	-2.87193200		H	-3.81624400	-0.17193600	1.78876500
	H	-1.21240200	-1.31639900	-1.91652700		H	-3.97189000	0.49781400	0.16886400
	H	-6.11704200	-1.50266500	0.90065700		H	-1.67074600	-4.66958200	-0.10074800
	H	-4.00852900	-2.69731500	1.94417000		H	-2.29954800	-3.43592800	-2.34620800
	H	-1.84423800	-2.28713900	0.48755200		H	-3.21074600	-0.99903900	-1.88909500
(*S*)-**1C6**	C	-5.59739300	-0.43593300	-0.43790000	(*S*)-**1C17**	C	5.29206800	-1.66719700	-0.41064800
	C	-5.36665700	0.79837300	-1.04221500		C	4.62541500	-1.87383400	-1.61682800
	C	-4.09813700	1.37250200	-0.99822100		C	3.33231000	-1.38743500	-1.79331000
	C	-3.04351200	0.72674500	-0.33758200		C	2.68109900	-0.69667000	-0.76153800
	C	-3.26153300	-0.51456600	0.28526100		C	3.33540200	-0.48783400	0.46512100
	C	-4.54705700	-1.08712000	0.21192300		C	4.65051100	-0.97199800	0.61616500
	C	-1.67928900	1.38532700	-0.29579800		C	1.27002800	-0.19311700	-0.99086200
	C	-0.54430800	0.38433200	-0.02078300		C	0.84424100	0.89277700	0.00002800
	C	-0.89409800	-0.42361500	1.23133400		C	1.16621300	0.42850900	1.42827500
	C	-2.15072000	-1.26788000	0.99167000		C	2.67915700	0.26626700	1.60551200
	O	-4.83129900	-2.30449000	0.76593700		O	5.36501100	-0.78657800	1.76735600
	N	0.79794400	0.97486100	0.05624800		N	-0.54974100	1.30357300	-0.23637100
	C	1.37664200	1.26725900	-1.25235700		C	-1.55762600	0.35068400	0.22840900
	C	0.94992700	2.07820200	1.00949900		C	-0.81813500	2.67699700	0.20683300
	C	2.35766500	2.14614600	1.61445600		C	-0.39524700	3.72383800	-0.82578500
	C	2.73747000	0.89515900	2.40583000		C	-0.66695300	5.15091000	-0.34884700
	C	1.91412800	0.01255700	-1.96938600		C	-2.80310900	0.37305900	-0.67698000
	C	2.93963600	-0.73578900	-1.17445600		C	-3.88267700	-0.55411600	-0.20774500
	S	4.54485600	-0.08344100	-0.91204400		S	-3.68681000	-2.29608600	-0.29435900
	C	4.99505300	-1.42818300	0.09622500		C	-5.23839900	-2.56859000	0.44594700
	C	3.96901500	-2.32283600	0.21507400		C	-5.86076800	-1.38863200	0.74058800
	C	2.79925400	-1.92509900	-0.50850100		C	-5.08583100	-0.24388500	0.36999600
	H	-6.57339700	-0.90971100	-0.46645200		H	6.30313000	-2.02777000	-0.25065800
	H	-6.17853600	1.30624400	-1.55476400		H	5.12217100	-2.40871800	-2.42110700
	H	-3.91439500	2.32928200	-1.47922400		H	2.81573200	-1.53844700	-2.73717400
	H	-1.50688400	1.91621200	-1.23794000		H	1.17489100	0.18822100	-2.01306100
	H	-1.67712100	2.14846700	0.49356400		H	0.57734900	-1.04238500	-0.91703100
	H	-0.51774100	-0.32370600	-0.85755200		H	1.45840400	1.77799900	-0.20247100
	H	-0.05466400	-1.07144000	1.50089500		H	0.78613400	1.14431500	2.16408100
	H	-1.06737600	0.25471900	2.07492200		H	0.67156200	-0.53086100	1.61978800
	H	-2.50791300	-1.65180500	1.95752600		H	2.87124800	-0.24571600	2.55897800
	H	-1.88151200	-2.15184500	0.39464200		H	3.14155900	1.26024300	1.69601000
	H	-4.03550400	-2.67502000	1.17586300		H	4.83847200	-0.28127900	2.40437300
	H	0.66381500	1.77400100	-1.92524300		H	-1.13487500	-0.65649800	0.20331600
	H	2.20850600	1.96167300	-1.10837200		H	-1.86509800	0.53504600	1.27122300
	H	0.70081300	3.05261100	0.55376300		H	-1.89443500	2.77776900	0.38003300
	H	0.23647300	1.92776800	1.82487500		H	-0.33239900	2.89246600	1.17491700
	H	3.09585800	2.31465900	0.82114600		H	0.67193400	3.61162200	-1.05371600
	H	2.40033000	3.02967600	2.26452200		H	-0.93504800	3.52554400	-1.75995300
	H	2.03944900	0.72760300	3.23532700		H	-0.11381300	5.37343600	0.57114200
	H	2.71649400	0.00906800	1.76721700		H	-0.36827300	5.88695000	-1.10245100
	H	3.74394700	0.98360600	2.82893300		H	-1.73230100	5.30279600	-0.13868500
	H	1.09297300	-0.67728200	-2.18815500		H	-3.22104100	1.38458600	-0.70819800
	H	2.32230100	0.32696700	-2.93746000		H	-2.48700800	0.12684700	-1.69719100
	H	5.98725400	-1.47561500	0.52455100		H	-5.58971700	-3.57843300	0.61049700
	H	4.03919000	-3.23509000	0.79710300		H	-6.83933700	-1.33176700	1.20427600
	H	1.87822100	-2.49759000	-0.53265000		H	-5.40837100	0.78060900	0.52258400
(*S*)-**1C7**	C	-4.80215500	0.15479700	-0.28323600	(*S*)-**1C18**	C	5.25503800	-0.73135700	0.02428000
	C	-4.45482000	0.75728000	-1.49097300		C	5.21620000	0.41676200	-0.76499600

	C	-3.14181100	1.16372900	-1.71803000		C	3.99737600	1.02544300	-1.05530500
	C	-2.15823200	0.98683600	-0.73476900		C	2.79581300	0.48637300	-0.57370000
	C	-2.49417100	0.38992500	0.49321800		C	2.81680000	-0.67695100	0.21489600
	C	-3.82311100	-0.03329100	0.69426600		C	4.06196500	-1.26443000	0.51564600
	C	-0.74089800	1.44973200	-1.00870500		C	1.48656300	1.16477400	-0.92566400
	C	0.28788200	0.75867000	-0.09996600		C	0.32933200	0.76806300	0.00723700
	C	-0.15451100	0.91444600	1.35552500		C	0.27387700	-0.75897500	0.10867700
	C	-1.46632500	0.15643100	1.58378900		C	1.54776600	-1.29278600	0.77051400
	O	-4.22059600	-0.64671500	1.85035300		O	4.16585700	-2.38119100	1.29845100
	N	1.68126500	1.16903500	-0.31631100		N	-0.97324200	1.34738000	-0.34422200
	C	2.31361200	0.50054300	-1.45280700		C	-1.48057100	0.99354700	-1.66964300
	C	1.94160300	2.60846000	-0.25124000		C	-1.08953800	2.78753400	-0.09904900
	C	3.35118300	2.92372600	0.26355200		C	-0.93543700	3.20548200	1.36617600
	C	3.58982000	2.44741300	1.69609400		C	-1.90427800	2.49986600	2.31459000
	C	3.13209500	-0.74312600	-1.03689300		C	-2.97814800	0.62411300	-1.64487400
	C	2.31977900	-1.78602900	-0.33268900		C	-3.24557300	-0.65986600	-0.91708000
	S	1.23742400	-2.84618600	-1.21728100		S	-3.66914500	-0.68843300	0.77792400
	C	0.60066800	-3.52456700	0.25423400		C	-3.68698900	-2.42577300	0.81312900
	C	1.19923800	-2.97401300	1.35295200		C	-3.37507400	-2.94801700	-0.41236800
	C	2.17768600	-1.98480000	1.01572600		C	-3.12726800	-1.94053600	-1.39632900
	H	-5.81520100	-0.18084400	-0.08586900		H	6.19210700	-1.21860900	0.27394800
	H	-5.21087700	0.89972500	-2.25773700		H	6.14181700	0.83956400	-1.14484300
	H	-2.86764900	1.62209000	-2.66423000		H	3.96764900	1.92725900	-1.66067900
	H	-0.50049500	1.27289800	-2.06290100		H	1.63284000	2.25001300	-0.91616200
	H	-0.68269200	2.53549500	-0.85614000		H	1.21588800	0.90082400	-1.95704100
	H	0.25532200	-0.31113000	-0.32184800		H	0.56736700	1.13927700	1.01006800
	H	0.62037500	0.52517200	2.02190600		H	-0.60419800	-1.06421800	0.68432800
	H	-0.29397900	1.97501300	1.59678600		H	0.17508500	-1.19892800	-0.89013400
	H	-1.88004600	0.44161700	2.56093400		H	1.57546700	-2.38491100	0.65278900
	H	-1.24602500	-0.91982900	1.64541400		H	1.49797800	-1.10463700	1.85331900
	H	-3.45783600	-0.77107000	2.43475500		H	3.29020100	-2.63703900	1.62396200
	H	1.57080400	0.20588200	-2.20945200		H	-1.33302800	1.81421400	-2.39040200
	H	2.99774300	1.19582900	-1.94860700		H	-0.93035200	0.13610700	-2.06175100
	H	1.79262300	3.09955700	-1.22870800		H	-0.37879800	3.36815000	-0.71629600
	H	1.21732700	3.05698200	0.43566700		H	-2.09167700	3.08328700	-0.43087600
	H	4.09588300	2.47410700	-0.40574400		H	0.09609000	3.04742700	1.70290300
	H	3.50053300	4.00930000	0.20097700		H	-1.09920700	4.28962200	1.40821800
	H	3.44976800	1.36551900	1.77554100		H	-1.72469200	1.42203400	2.33908200
	H	4.60510100	2.68450300	2.03173900		H	-1.80725400	2.88515500	3.33547700
	H	2.88772900	2.92416600	2.39070700		H	-2.94293100	2.65101300	1.99709500
	H	3.60726400	-1.16052800	-1.93308200		H	-3.54927600	1.44735100	-1.20329400
	H	3.93189500	-0.42288000	-0.36151800		H	-3.32303500	0.51704800	-2.67999400
	H	-0.15153300	-4.30091900	0.21171500		H	-3.92739000	-2.94995900	1.72850000
	H	0.95767300	-3.26388200	2.36955000		H	-3.32874100	-4.01298000	-0.61201800
	H	2.74821800	-1.42428800	1.74770600		H	-2.86689300	-2.15386900	-2.42784600
(*S*)-**1C8**	C	5.02379600	-1.90892200	0.22187400	(*S*)-**1C19**	C	5.52632300	-1.25601800	0.56676600
	C	4.35864900	-2.14904100	1.42273500		C	5.24980100	-0.46187300	1.67826900
	C	3.17512300	-1.47450300	1.71411200		C	4.03966800	0.22203300	1.76609700
	C	2.64465600	-0.54005000	0.81359900		C	3.09205500	0.13384000	0.73640200
	C	3.30694500	-0.28059800	-0.39872600		C	3.36006900	-0.65310200	-0.39700600
	C	4.49291200	-0.98682700	-0.68222500		C	4.58082600	-1.35494100	-0.45557300
	C	1.36503700	0.18968800	1.16953100		C	1.79226000	0.90292700	0.86478400
	C	0.64422800	0.76466900	-0.06167100		C	0.69366000	0.39778300	-0.07345800
	C	1.64237400	1.59219700	-0.87449300		C	1.25997900	0.27075400	-1.49533500
	C	2.76189200	0.69885600	-1.41992000		C	2.36610300	-0.78834700	-1.53385400
	O	5.17912600	-0.81077100	-1.85186400		O	4.90080900	-2.16390300	-1.51072800
	N	-0.58741800	1.51124100	0.23677000		N	-0.52590500	1.21278600	0.05367700
	C	-1.72760400	0.63463800	0.50220900		C	-1.73238200	0.46538000	-0.30347900
	C	-0.45020500	2.58911500	1.22322000		C	-0.46336500	2.53190200	-0.58662500
	C	-1.41914300	3.74720100	0.95770500		C	-1.32958800	3.56360100	0.13944400
	C	-1.16216700	4.45243700	-0.37378300		C	-1.28202000	4.93653200	-0.53164100
	C	-2.41303800	0.17657700	-0.79923400		C	-2.26084400	-0.36211900	0.88279200
	C	-3.55953500	-0.75670600	-0.55561500		C	-3.46365300	-1.18353600	0.53124400
	S	-3.29025800	-2.41142800	-0.03755600		S	-5.02030900	-0.43225600	0.22831700
	C	-4.99979300	-2.72484700	0.05734000		C	-5.75776900	-1.96555700	-0.13864700
	C	-5.72542800	-1.61932500	-0.28647400		C	-4.85546700	-2.98625300	-0.03543700
	C	-4.90354100	-0.50005800	-0.63275800		C	-3.55011700	-2.53819600	0.34357800
	H	5.94234400	-2.42773800	-0.03303000		H	6.45679500	-1.80772800	0.47921100
	H	4.76367100	-2.87017000	2.12681900		H	5.97892400	-0.38621600	2.47980200
	H	2.65029600	-1.66872100	2.64542400		H	3.81833700	0.83116600	2.63817100
	H	0.70593300	-0.48830100	1.72185000		H	1.43676400	0.85807000	1.89971100
	H	1.60554300	1.01292800	1.85489600		H	1.98825700	1.96316100	0.65602300
	H	0.33979900	-0.07952500	-0.69267100		H	0.41961800	-0.61052900	0.26021500

	H	1.12611900	2.08966800	-1.70101500		H	0.47074900	0.00192200	-2.20471500
	H	2.07708300	2.37509600	-0.24205100		H	1.66838400	1.23757300	-1.81165900
	H	3.57030100	1.33824300	-1.80106500		H	2.88605200	-0.72412800	-2.49987200
	H	2.38370500	0.13829200	-2.28754100		H	1.91022100	-1.78900500	-1.49993600
	H	4.72580700	-0.15707700	-2.40498500		H	4.16158200	-2.19522500	-2.13612700
	H	-1.43959400	-0.24847900	1.09589300		H	-2.50770200	1.17466800	-0.60528400
	H	-2.46324500	1.17924700	1.09983400		H	-1.56938600	-0.20153600	-1.16622500
	H	-0.59104000	2.22062300	2.25360600		H	0.57290600	2.88133600	-0.56797900
	H	0.57046700	2.97890700	1.17005400		H	-0.75536500	2.48723200	-1.65068700
	H	-2.45243700	3.37863400	0.98774500		H	-2.36824000	3.21246200	0.18228000
	H	-1.32592800	4.46327100	1.78418800		H	-0.98282600	3.63427800	1.17775500
	H	-1.25641200	3.75223000	-1.20890600		H	-1.65025000	4.88647000	-1.56301100
	H	-1.87126100	5.27140800	-0.53533200		H	-1.89787900	5.66483800	0.00614000
	H	-0.15031800	4.87410300	-0.40659200		H	-0.25775900	5.32630800	-0.56504800
	H	-2.78924800	1.05915500	-1.32608900		H	-1.47979100	-1.04492100	1.23289700
	H	-1.67150400	-0.29472300	-1.45433600		H	-2.47927500	0.32393200	1.70907000
	H	-5.35550600	-3.70134600	0.35777800		H	-6.80621900	-2.01305300	-0.40111000
	H	-6.80960500	-1.59904700	-0.29653800		H	-5.10826200	-4.02454900	-0.21961500
	H	-5.29289300	0.46663100	-0.93407800		H	-2.69906300	-3.19757000	0.47708000
(*S*)-**1C9**	C	5.28120700	-0.72694800	-0.02535000	(*S*)-**1C20**	C	-5.35412700	-1.41208800	0.77016300
	C	5.17362200	0.28392300	-0.97868000		C	-4.76149600	-1.13141900	1.99985800
	C	3.92704600	0.82198100	-1.28989600		C	-3.46718200	-0.61910100	2.05199100
	C	2.76632700	0.34736800	-0.66270200		C	-2.74012400	-0.39222000	0.87484600
	C	2.85613400	-0.67783700	0.29480500		C	-3.31956100	-0.67879200	-0.37333600
	C	4.12903900	-1.19404700	0.60998800		C	-4.63740900	-1.17738500	-0.40484600
	C	1.42414900	0.94142000	-1.04048100		C	-1.32538500	0.14350800	0.97034200
	C	0.34232700	0.70753400	0.02790800		C	-0.83918900	0.79150500	-0.33710200
	C	0.30992300	-0.78128900	0.38292800		C	-1.08361500	-0.18202400	-1.49206200
	C	1.63554200	-1.21209500	1.01922500		C	-2.58388100	-0.43290400	-1.67642800
	O	4.30182300	-2.17235600	1.54985700		O	-5.27973000	-1.45609700	-1.57968500
	N	-0.98917400	1.21989600	-0.31923300		N	0.55011600	1.27565300	-0.30880900
	C	-1.54084200	0.74120100	-1.58429500		C	1.54160400	0.28692800	0.11306800
	C	-1.13339600	2.66693600	-0.14177200		C	0.69508000	2.56227300	0.38338900
	C	-1.33728200	3.07553900	1.31907500		C	0.34706400	3.75601600	-0.50830400
	C	-1.49693400	4.58792300	1.47767500		C	0.49287400	5.08917200	0.22607200
	C	-3.05622600	0.46586800	-1.48102400		C	2.88327000	0.49882600	-0.61323600
	C	-3.37976900	-0.63702800	-0.52071100		C	3.94777100	-0.45330500	-0.15972800
	S	-3.15311200	-2.31779100	-0.96627900		S	3.86176000	-2.16447500	-0.54017700
	C	-3.58765300	-2.86473000	0.62753700		C	5.32265700	-2.49716500	0.34579600
	C	-3.89083000	-1.81254400	1.44539200		C	5.83663700	-1.35868000	0.89910600
	C	-3.76898500	-0.54584500	0.78984900		C	5.05091900	-0.19749200	0.61188100
	H	6.24132000	-1.15600500	0.24285000		H	-6.36458300	-1.80209500	0.70168200
	H	6.06737500	0.65580700	-1.47134900		H	-5.31680600	-1.30643700	2.91681500
	H	3.84330500	1.61768900	-2.02502200		H	-3.00803900	-0.39016200	3.00975000
	H	1.54647400	2.01196000	-1.23614500		H	-1.26528300	0.85606100	1.79963800
	H	1.09227500	0.49143700	-1.98555500		H	-0.65289600	-0.68549700	1.22696900
	H	0.64747900	1.24584100	0.93282400		H	-1.46143900	1.67470200	-0.52384000
	H	-0.51918000	-0.98124800	1.06801300		H	-0.65538000	0.22355800	-2.41376400
	H	0.13161500	-1.37421400	-0.52136300		H	-0.57829100	-1.13358000	-1.28790500
	H	1.66945000	-2.30984600	1.05691100		H	-2.71919200	-1.28687500	-2.35434700
	H	1.66064900	-0.87110400	2.06479500		H	-3.03365000	0.43256200	-2.18521500
	H	3.44934200	-2.39647900	1.95210000		H	-4.71178500	-1.22204200	-2.32892600
	H	-1.36829100	1.45186000	-2.40886300		H	1.71629800	0.29961900	1.20185300
	H	-1.04437900	-0.18942200	-1.86808100		H	1.17435600	-0.71273900	-0.13127800
	H	-0.27628700	3.22158700	-0.56436000		H	0.08931100	2.59910500	1.30551100
	H	-2.01177200	2.98811600	-0.71168200		H	1.73736300	2.66284400	0.70314500
	H	-2.22406800	2.55679700	1.70360000		H	1.00143400	3.73269100	-1.38848000
	H	-0.48993000	2.73378700	1.92557400		H	-0.67957000	3.65487100	-0.88128400
	H	-0.60752400	5.11975800	1.11997700		H	0.24994400	5.93338200	-0.42752700
	H	-2.35582200	4.95832500	0.90557100		H	-0.17433000	5.13797900	1.09457400
	H	-1.65061700	4.86502200	2.52575300		H	1.51776900	5.23240700	0.58840500
	H	-3.56931800	1.37446400	-1.14845600		H	2.70607100	0.41070200	-1.69119800
	H	-3.43657100	0.23401700	-2.48283400		H	3.24592200	1.51580800	-0.43179200
	H	-3.60613000	-3.92289000	0.85154700		H	5.70682500	-3.50714500	0.39697600
	H	-4.19325900	-1.93139200	2.48002400		H	6.74529800	-1.34268800	1.49067200
	H	-3.95710700	0.40637500	1.27356800		H	5.29405600	0.79985100	0.96273000
(*S*)-**1C10**	C	-5.33641900	-1.27881500	-0.81131500	(*S*)-**1C21**	C	5.07010300	-1.80101900	0.53434200
	C	-5.11142800	-0.23456000	-1.70660800		C	4.38227200	-1.83032000	1.74596600
	C	-3.94737700	0.52516200	-1.61779900		C	3.18120000	-1.13853100	1.88548600
	C	-2.99515600	0.26069500	-0.62316000		C	2.65551300	-0.39672600	0.81835200
	C	-3.21146700	-0.78248000	0.29401000		C	3.33974800	-0.35159100	-0.40881000
	C	-4.38613600	-1.55223500	0.17423200		C	4.54416400	-1.07245600	-0.53437500

	C	-1.74754200	1.11929300	-0.55253100		C	1.35790600	0.36215100	1.01120900
	C	-0.61915900	0.48406100	0.26298000		C	0.65433100	0.68883200	-0.31678500
	C	-1.17825000	-0.00405900	1.60749600		C	1.65710300	1.36976700	-1.25059500
	C	-2.20847100	-1.11409800	1.38118600		C	2.80009000	0.41178000	-1.60307300
	O	-4.65610700	-2.59933800	1.01210900		O	5.25583800	-1.09890600	-1.70190300
	N	0.54448700	1.38188500	0.35033600		N	-0.59162600	1.46003900	-0.18350400
	C	1.79664000	0.64951300	0.53795400		C	-1.72381900	0.63046100	0.23057000
	C	0.39672600	2.50399000	1.28593500		C	-0.48128300	2.70548000	0.58401500
	C	1.20118200	3.73429100	0.85177800		C	-1.46515400	3.77092200	0.09449900
	C	0.72770500	4.32158400	-0.47756200		C	-1.36357300	5.06728200	0.89873300
	C	2.37782000	0.15664500	-0.80081600		C	-2.39881600	-0.05609900	-0.97205500
	C	3.64366300	-0.62720200	-0.63292000		C	-3.55055400	-0.92665000	-0.57145000
	S	3.62719600	-2.25286700	0.02753800		S	-3.29162600	-2.46897100	0.22406500
	C	5.36118900	-2.37405900	-0.06526200		C	-5.00306200	-2.74614500	0.37904200
	C	5.91091800	-1.22986300	-0.57062500		C	-5.72171700	-1.71052300	-0.14824400
	C	4.93167600	-0.23654100	-0.89064700		C	-4.89293200	-0.67534300	-0.68623800
	H	-6.23052000	-1.89154400	-0.86568800		H	6.00301700	-2.33826200	0.39692300
	H	-5.84437300	-0.02189100	-2.47946100		H	4.78351600	-2.40123100	2.57834000
	H	-3.76671500	1.33204600	-2.32267600		H	2.63907800	-1.16859800	2.82658200
	H	-1.38971600	1.33364300	-1.56518300		H	0.69778900	-0.21452000	1.66754800
	H	-2.01132400	2.08948600	-0.11118200		H	1.57249900	1.30141600	1.53758900
	H	-0.27911800	-0.40100400	-0.28835500		H	0.37138200	-0.26136400	-0.78682100
	H	-0.37414100	-0.37641000	2.25031500		H	1.14925900	1.69879100	-2.16233200
	H	-1.65332000	0.83411500	2.13016800		H	2.06809700	2.26248500	-0.76464800
	H	-2.73036700	-1.31245700	2.32740600		H	3.60648400	0.98411100	-2.08195400
	H	-1.68482800	-2.04603700	1.12109900		H	2.44760400	-0.30644800	-2.35807200
	H	-3.91413700	-2.73369000	1.62063400		H	4.79514400	-0.58378000	-2.38102200
	H	2.53133200	1.31221200	1.00241100		H	-1.42865400	-0.13180900	0.96985800
	H	1.67299600	-0.20511700	1.22397400		H	-2.46785000	1.26262500	0.72213000
	H	-0.66007200	2.78375000	1.32647200		H	-0.62871400	2.53895100	1.66573000
	H	0.68346300	2.21847600	2.31234800		H	0.53336400	3.09734900	0.46864900
	H	1.12059400	4.48907100	1.64441700		H	-1.26484000	3.96367900	-0.96661900
	H	2.26548700	3.47540900	0.78393600		H	-2.49004300	3.38341500	0.15193600
	H	0.79149500	3.57567400	-1.27519500		H	-0.35556600	5.49417400	0.83695800
	H	-0.31639800	4.65060700	-0.41263900		H	-2.06735300	5.82099900	0.53060500
	H	1.33264600	5.18635500	-0.77049700		H	-1.58490300	4.89458300	1.95843500
	H	1.62616800	-0.44273900	-1.32718200		H	-2.76711700	0.71760200	-1.65319700
	H	2.58972900	1.02650700	-1.43046900		H	-1.65298300	-0.63951100	-1.52402900
	H	5.85899000	-3.27943100	0.25546500		H	-5.36546300	-3.65314200	0.84411900
	H	6.97728700	-1.09552500	-0.71464600		H	-6.80573600	-1.68291300	-0.15751200
	H	5.17045700	0.73795200	-1.30324800		H	-5.27642600	0.22828700	-1.14803000
(*S*)-**1C11**	C	-5.46482800	-0.65548000	0.05685800	(*S*)-**1C22**	C	5.24529700	-1.66429500	0.62187300
	C	-5.31188200	0.40575100	0.94703800		C	4.56667600	-1.66154100	1.83911400
	C	-4.04029000	0.88826800	1.24853400		C	3.33732700	-1.01634300	1.95345700
	C	-2.90073200	0.30798900	0.67374100		C	2.77383200	-0.35180800	0.85501700
	C	-3.03683500	-0.76852600	-0.21955300		C	3.44965600	-0.33716900	-0.37739300
	C	-4.33292300	-1.22890200	-0.52582400		C	4.68183300	-1.01350600	-0.47734400
	C	-1.53101300	0.84468100	1.03749300		C	1.44459400	0.35759400	1.01895300
	C	-0.45580000	0.49300500	-0.00597800		C	0.72431100	0.59125700	-0.31972600
	C	-0.49792000	-1.01557700	-0.25960000		C	1.69414300	1.27181800	-1.28820900
	C	-1.83837800	-1.41523100	-0.88631600		C	2.87642300	0.34878700	-1.60236200
	O	-4.54935000	-2.25321300	-1.40553800		O	5.38531000	-1.06819200	-1.64879100
	N	0.89727300	0.97606300	0.31911800		N	-0.55412500	1.31187700	-0.21803900
	C	1.39281700	0.57169600	1.63475000		C	-1.65210500	0.45269200	0.22694300
	C	1.05985700	2.41331900	0.06642400		C	-0.50010400	2.59188800	0.49648300
	C	1.31955000	2.73694300	-1.40600700		C	-1.52853800	3.59179400	-0.03765700
	C	1.45167700	4.24068000	-1.64981100		C	-1.48905200	4.92177900	0.71553100
	C	2.92624400	0.46602200	1.67002700		C	-2.28483400	-0.31904100	-0.94664800
	C	3.50216900	-0.63328900	0.82941700		C	-3.38546300	-1.23747300	-0.51030300
	S	3.25956100	-0.71961900	-0.90491500		S	-4.95331600	-0.61292100	-0.03002800
	C	4.26396600	-2.13653900	-1.03142100		C	-5.53627500	-2.20552600	0.36122500
	C	4.73488100	-2.52847000	0.19029800		C	-4.57715400	-3.15262600	0.13755700
	C	4.30352700	-1.66581200	1.24665400		C	-3.35350700	-2.59884900	-0.35644600
	H	-6.44460800	-1.04365100	-0.20237600		H	6.19915300	-2.16826200	0.50360400
	H	-6.18938500	0.85988900	1.39819400		H	4.99730100	-2.17185900	2.69580400
	H	-3.92084400	1.72247200	1.93434300		H	2.80217700	-1.02327300	2.89899600
	H	-1.59688100	1.92825400	1.17909100		H	0.81183900	-0.21694900	1.70355600
	H	-1.23319700	0.42478300	2.00729200		H	1.61876400	1.32831900	1.50129500
	H	-0.73134500	0.98338700	-0.94683800		H	0.48338200	-0.39129800	-0.74405700
	H	0.32397700	-1.30552400	-0.91845200		H	1.16986500	1.53703000	-2.21134300
	H	-0.36418000	-1.55574000	0.68505900		H	2.06737500	2.20252300	-0.84494100
	H	-1.93178500	-2.50978200	-0.85223000		H	3.65824000	0.93261600	-2.10755800
	H	-1.83187700	-1.14449900	-1.95258000		H	2.55386100	-0.41608600	-2.32422200

	H	-3.70516600	-2.55944600	-1.76913000		H	4.89070500	-0.62037000	-2.35139000
	H	1.07500600	1.26505900	2.43171500		H	-1.33516300	-0.26525100	1.00065700
	H	0.97839000	-0.40796400	1.88327800		H	-2.42250800	1.07833600	0.68555400
	H	0.18896900	2.98992300	0.42361100		H	-0.64261900	2.46459900	1.58426800
	H	1.91415900	2.76884600	0.65049500		H	0.49668100	3.02277100	0.36536600
	H	2.23447900	2.22022200	-1.71888400		H	-1.33236800	3.75181000	-1.10495300
	H	0.50821600	2.33697900	-2.02607700		H	-2.53535000	3.16121300	0.03201300
	H	2.27263800	4.66736800	-1.06139800		H	-0.50097000	5.39098300	0.64020000
	H	1.65086800	4.45745500	-2.70444300		H	-2.22420100	5.62820600	0.31624300
	H	0.53384300	4.77021500	-1.36864900		H	-1.70744600	4.78055700	1.78050100
	H	3.36137200	1.42978900	1.37864600		H	-2.64586800	0.40906700	-1.68158900
	H	3.23690900	0.28813000	2.70544000		H	-1.52044800	-0.92412000	-1.44505100
	H	4.41692900	-2.60779600	-1.99366300		H	-6.54664600	-2.33836600	0.72438100
	H	5.36531300	-3.39821200	0.33979100		H	-4.73405000	-4.21103200	0.31388700
	H	4.58459000	-1.79615100	2.28654100		H	-2.47461800	-3.18862900	-0.59455700

Table S8. Major orbital contributions, excitation energies, wavelengths, and rotatory strengths of the first seven excited states of (*S*)-**2**.

Transition States	Main Transition Orbitals (hole → electron)	Contribution	Wavelength (nm)	Rotatory Strength (10^{-40}cgs)
1	MO52→MO53	97.95%	252.2	8.157
	MO50→MO54	17.45%		
2	MO51→MO53	56.06%	245.9	1.260
	MO52→MO54	25.14%		
	MO50→MO54	7.33%		
3	MO51→MO53	17.10%	238.4	-2.786
	MO52→MO54	73.68%		
4	MO50→MO53	33.63%	217.1	5.339
	MO51→MO54	64.49%		
5	MO52→MO55	79.54%	203.8	5.098
	MO52→MO56	18.75%		
	MO50→MO53	16.84%		
6	MO50→MO54	50.57%	194.1	-13.542
	MO51→MO53	17.45%		
	MO51→MO54	10.50%		
	MO50→MO53	45.29%		
	MO50→MO54	20.39%		
7	MO51→MO53	6.34%	192.8	3.198
	MO51→MO54	21.80%		
	MO51→MO55	2.37%		

Table S9. Atomic contributions of non-hydrogen atoms to the first seven excited states of (*S*)-**2**.

Atoms	Transition State 1		Transition State 2		Transition State 3		Transition State 4		Transition State 5		Transition State 6		Transition State 7	
	Hole	Electron	Hole	Electron	Hole	Electron	Hole	Electron	Hole	Electron	Hole	Electron	Hole	Electron
1(O)	0.04%	0.36%	9.68%	2.93%	2.97%	5.28%	11.18%	4.27%	0.04%	0.26%	5.12%	4.21%	5.42%	3.00%
3(C)	0.10%	5.33%	11.13%	12.13%	3.25%	18.33%	13.09%	15.68%	0.10%	0.30%	7.79%	15.30%	8.19%	12.24%
4(C)	0.37%	17.22%	10.44%	12.98%	2.52%	9.11%	13.44%	10.76%	0.37%	0.87%	14.67%	12.35%	16.94%	10.66%
5(C)	1.22%	16.82%	7.71%	14.30%	2.07%	12.01%	10.17%	12.99%	1.22%	1.17%	16.58%	10.43%	15.33%	16.99%
6(C)	5.30%	2.57%	2.51%	2.33%	4.19%	2.11%	1.68%	2.21%	5.30%	4.70%	2.98%	1.78%	2.67%	2.96%
9(C)	6.28%	0.54%	1.68%	0.51%	4.89%	0.48%	0.29%	0.50%	6.28%	3.06%	0.46%	0.44%	0.43%	0.65%
11(N)	53.40%	0.09%	10.57%	0.09%	43.92%	0.09%	0.50%	0.09%	53.40%	1.96%	0.94%	0.08%	0.83%	0.14%
12(C)	6.70%	0.20%	1.45%	0.16%	5.38%	0.12%	0.06%	0.14%	6.70%	7.52%	0.12%	0.10%	0.10%	0.31%
16(C)	6.58%	0.05%	1.22%	0.06%	5.56%	0.08%	0.13%	0.07%	6.58%	9.12%	0.24%	0.06%	0.21%	0.28%
20(C)	1.60%	0.53%	0.51%	0.48%	1.44%	0.44%	0.36%	0.45%	1.60%	3.25%	0.48%	0.53%	0.52%	0.58%
23(C)	0.31%	2.77%	1.75%	2.05%	0.51%	1.40%	2.14%	1.68%	0.31%	1.83%	2.54%	2.05%	2.94%	1.69%
26(C)	0.70%	5.72%	13.35%	12.44%	4.23%	18.58%	15.46%	15.96%	0.70%	0.79%	9.62%	16.22%	9.30%	11.55%
28(C)	0.28%	18.97%	7.76%	15.17%	2.13%	11.70%	10.30%	13.18%	0.28%	0.37%	11.90%	16.06%	13.01%	11.07%
30(C)	0.48%	17.11%	10.54%	13.46%	2.20%	10.13%	13.13%	11.56%	0.48%	0.23%	17.14%	9.84%	14.69%	15.15%

Table S10. Hole–electron analysis parameters (D, Sr, H, Δσ, t, HDI, and EDI) for the first seven excited states of (*S*)-**2**.

**Transition States**	***D*** **(Å)**	***S*r (a.u.)**	***H*** **(Å)**	**Δ*σ*** **(Å)**	***t*** **(Å)**	**HDI**	**EDI**
1	4.764	0.203	1.930	0.361	3.417	17.68	9.35
2	0.900	0.844	2.436	-0.676	-0.850	7.54	7.73
3	3.873	0.465	2.371	-0.571	2.076	14.66	8.49
4	0.332	0.920	2.077	0.027	-1.023	7.96	7.88
5	0.387	0.387	2.471	1.443	-0.997	17.74	3.32
6	0.244	0.868	2.095	-0.011	-1.099	7.44	7.78
7	0.189	0.887	2.172	0.167	-1.081	7.45	7.60

Note: *D* denotes the distance between the centroids of the hole and electron distributions, whereas *S*r quantifies their spatial overlap. The *t* index characterizes the degree of spatial separation between the hole and electron. *H* describes their average spatial extent, and Δ*σ* represents the difference between the spatial extents of the electron and hole distributions. HDI and EDI quantify the degrees of hole and electron delocalization, respectively, with lower values indicating greater delocalization.
